# PROJECTA: An Art-Based Tool in Trauma Treatment

**DOI:** 10.3389/fpsyg.2020.568948

**Published:** 2020-12-18

**Authors:** Marián López Fernández-Cao, Celia Camilli-Trujillo, Laura Fernández-Escudero

**Affiliations:** ^1^Department of Languages, Arts and Physical Education, Complutense University of Madrid, Madrid, Spain; ^2^Department of Research and Psychology in Education, Complutense University of Madrid, Madrid, Spain; ^3^Pre-doctoral Researcher at Project Art, Art Therapy, Trauma and Emotional Memory (HAR2015-5 69115-R), Department of Languages, Arts and Physical Education, Complutense University of Madrid, Madrid, Spain

**Keywords:** art therapy, therapy, artistic images, emotions, trauma, validation, tool, mixed methods research

## Abstract

Artistic images, of a universal nature and validated by global culture, are carriers of an emotional potential that can be used for therapeutic purposes in cultural centers as well as in clinical spaces. Esthetic studies reveal the mobilizing power in their contemplation and the capacity to bring out personal stories with healing potential. The general objective of this paper is to design and validate the PROJECTA instrument, consisting of the therapeutic use of artistic images to approach trauma or difficult conditions in therapy, by means of the association between art images and emotions, feelings or states of mind related with a traumatic experience. A mixed approach of investigation with a concurrent triangulation design has been used for the integration of qualitative and quantitative results, where qualitative research outweighs quantitative, but both have been QUAL → quan sequentially developed. The study was carried out in two phases. The first was a systematic review of the literature. The second, divided into five steps, consisted of the validation of artworks and the associated emotions with the participation of students, experts in art therapy, psychologists and educators through focus groups, interviews and an online questionnaire. The analysis techniques were qualitative and quantitative. A set of 220 artistic images linked to emotional states were proposed by experts in education, psychology and art therapy, and validated through an online questionnaire. The respondents included 228 students and professionals and for 64.55% of these images a consensus of over 80% was obtained. These images and their associated emotions were again reviewed by a confirmatory focus group. Finally, 92 artistic works were linked to primary, secondary and tertiary emotions such as love, joy, victory, surprise, balance, sense of humor, anger, sadness, fear, and emptiness. In addition, there are mobilizing images that have not been related to any emotion because of the polysemy of their meanings. It concludes that PROJECTA is a visual art therapy tool that provides professionals with a resource that helps facilitate the identification, expression or demonstration of emotions or feelings related with trauma, and in challenging situations of vulnerability, psychological discomfort or post-traumatic stress.

## Introduction

The study of the traumatic origins of emotional distress began during the last decades of 19th Century (Van der Kolk, 2003), with authors like Pierre Janet and later, Freud. Afterward, it became the focus of attention again through authors such as Kardiner (1941) in his work related to the psychological effects on the veterans of the First World War, “The traumatic neuroses of War.” In this work, he related, in the same way as Janet did decades before, how patients remain in a permanently altered state with regard to themselves and the outside world. It was not until 30 years later, with the Vietnam War and the emergence of the women’s emancipation movement, that the significance of trauma was discovered. Subsequent studies led to the acceptance of post-traumatic stress disorder (PTSD) by the American Psychiatric Association in 1980. Thus, little by little, the mechanisms that human beings use to deal with the atrocities they have lived through or seen have been accepted and studied. This process often includes denial, dissociation or repression, and impedes those affected from leading a stable life adapted to the standards of human well-being.

The common denominator of psychological trauma, according to the Comprehensive Textbook of Psychiatry (2000), is the feeling of intense fear, fragility, loss of control and threat of annihilation. In this sense, trauma is extraordinary, not because it is a rare occurrence, but because it exceeds the normal capacity to adapt to life. The human being is left with a feeling without language, pre-verbal. His/her body becomes a symptom and relives terror, rage or impotence, unleashing the impulse to fight, freeze or flee, to act or not to act, in ways and with feelings that are impossible to understand and difficult to articulate (Van der Kolk, 2015). Her/his body will probably “keep the score” –in reference to the title of Van der Kolk’s work- for the rest of his/her existence (Cao, 2017). According to Foa (2006), posttraumatic stress disorder may be underpinned by dysfunctional cognition involving negative thoughts about the world, negative thoughts about self -including feelings of incompetence and weakness-, and self-blame (guilt).

After one hundred years of study, some characteristics of traumatic memories could be summarized as follows, after Van der Kolk (2003):

(1) They are primarily imprinted in sensory and emotional modes, although a semantic representation of the memory may co-exist with sensory flashbacks (Van der Kolk and Fisler, 1995).
(2) These sensory experiences often remain stable over time and unaltered by other life experiences (Janet, 1893; Van der Kolk and Van der Hart, 1991).
(3) They may return, triggered by reminders, at any time during a person’s life, with a vividness as if the subject is living the experience all over again (DSM IV).
(4) These sensory imprints tend to occur in a mental state in which victims may be unable to precisely articulate what they are feeling and thinking (Blank, 1985; Rivers, 1918).

Since that time, many studies have been carried out on how to help people suffering from PTSD (Van der Kolk, 1994; Rothschild, 2000; Neimeyer, 2001; Steel and Raider, 2001; Greenwald, 2003; Meichenbaum, 2003; Omaha, 2004; Follette and Ruzek, 2006) and many approaches have been taken from various domains. PTSD treatments must recognize the diversity, complexity and transformability of posttraumatic conditions (Wilson et al., 2001) that call for a holistic approach. One approaches is Cognitive Behavioral Therapy (CBT), which includes Exposure Therapy (ET), Anxiety Management (SIT), Cognitive Therapy (CT), Prolonged Exposure Therapy (PE) (Foa, 2006) or Cognitive Processing Therapy (CPT). Patients who received CBT had a greater decrease in intrusive memories at posttreatment than those who received supportive counseling (Ehlers and Clark, 2008). From all those it is important to highlight that main behavioral interventions are (1) prioritizing safety (2) addressing threats (3) stimulating the will to live and positive dispositional qualities (4) identity work (5) psychoeducation, (6) stress inoculation, (7) trauma narration, and (8) advocacy, social justice and reconnecting to social networks (Kira et al., 2015). Other options are Brief Eclectic Psychotherapy (BEP), which combines elements of cognitive behavioral therapy with a psychodynamic approach, Eye Movement Desensitization and Reprocessing (EMDR) therapy (Shapiro, 2001), and Narrative exposure therapy (Schauer et al., 2011), among others.

Due to the importance of integrating the experience or experiences lived in the treatment of trauma, the perception of images and sensory elements (smell, sound, and body sensations) take on special importance since they allow the integration of the functions of the left and right hemisphere of the brain that have been disarticulated and fragmented in the traumatic process (McNamee, 2003, 2005). As Talwar (2007) states, in trauma treatment it is not the verbal account of the event that is important, but the non-verbal memory of the fragmented sensory and emotional elements of the traumatic experience (Van der Kolk, 2003).

It is at precisely this point that art, both in its production of works and in its consumption and perception of them, can play a fundamental role in helping in the therapies used to confront the trauma. The perception of culturally validated works of art, legitimized by society as a whole, can, in their empathic potential which we will now discuss, help people affected by PTSD to make their experience visible in a coherent way, to legitimize, share and organize it.

### Art, Aesthetic Perception, Empathy. From Einfühlung to Aesthetic Shock

Segal (1955) pointed out that the aesthetic pleasure produced by the contemplation of the work of art, would be defined by the observer’s degree of identification with it, in such a way that the apprehension of the work of art is not only intellectual or aesthetic, but also affective and unconscious (Loza, 2006).

Aesthetic experience is our ongoing sense of the quality of the formal organization of our world, and this term applies to both our perception of the external world and our subjective experience. We can say that our aesthetic experience deeply influences our sense of self (Hagman and Press, 2010).

Depew refers to the term “empathy” from the late-nineteenth-century German coinage of *Einfühlung*, which literally means in-feeling. In its first use, in 1873, by German psychologist Robert Vischer, *Einfühlung* refers to the placing of human feelings into inanimate things, plants, animals, or other humans in a specific way (Depew, 2005). This term was subsequently linked to empathy by Lipps (1979), who forged this link when, around 1910, he referred to *empatheia* as the Greek equivalent of *Einfühlung*. *Empatheia* referred to an intense passion or state of emotional undergoing (Depew, 2005).

Other authors highlight the relationship of the aesthetic dimension with the concept of “vitality affect” and the procedural memory, pointed out by Stern, which links aesthetic emotion with the early relationship of the primary experience of the infant and his or her caregiver. Press refers to Daniel Stern (1985) description of vitality affects that poignantly captures qualitative dimensions of experience: “surging, fading away, fleeting, explosive, crescendo, decrescendo, bursting, drawn out, and so on” (p. 54). These vitality affects filter and flavor subjective exchanges, commingling emotional response and aesthetic qualities (Hagman and Press, 2010), rooted in our development of procedural memory, which is fueled with vitality affects and aesthetic qualities (Hagman and Press, 2010).

At this point, it is important to emphasize what Donald Kuspit calls Aesthetic Shock, differentiating it from “aesthetic pleasure,” as a central element of artistic contemplation coupled with empathy, in-feeling (*Einfühlung*) and vitality, pointing it out as the opposite of the state of alienation:

Aesthetic shock, with has been called its defamiliarizing effect, makes one aware that there is a World of extraordinary meaning and vitality beyond ordinary meaning and drive. It is the alternative to alienation in the everyday. Indeed, aesthetic shock transforms alienation into transcendence (Kuspit, 2006, p. 348).

Art, “enacting conflicts,” as opposed to alienation, allows awareness to be raised. In a safe space, such as the museum, workshop or therapy space, it has the opportunity to achieve “successful symbolization.” Thus, the creation “convincingly mixes concrete thinking and symbolic illusion, serves to represent seemingly unrepresentable feelings” (Kuspit, 2006 p. 346). As the philosopher Jacques Maritain (1953) states, art becomes “that intercommunication between the inner being of things and the inner being of the human self which is a kind of divination” (p. 3). In general, Western Aesthetics speaks of “aesthetic pleasure” rather than aesthetic shock, but “aesthetic pleasure is an aftereffect of aesthetic shock” (Kuspit, 2006, p. 351). Aesthetic pleasure becomes a consequence of confrontation, when contemplating a work of art, with a different vital state, with an alternative vision that reveals a part of oneself and its relationship with the world. Through aesthetic emotion:

one gains insight into their logic. Subsumed as aesthetic factors, our emotions, however, strong, no longer victimize us, but can be analyzed and above all criticized, dissipating their painful effect. Ironically mastered by aesthetic appropriation –explored by aesthetic consciousness, which is a species of critical consciousness- they become pure performances, uncannily felt rather than overwhelming and blatant (Kuspit, 2006, p. 353).

The aesthetic object,

is perceived as real without referring to the real (…) The aesthetic object is nothing more than the sensuous in all its Glory, whose form, ordering it, manifests plenitude and necessity, and which carries with itself and immediately reveals the meaning that animates it (Dufrenne, 1987, p. 5).

### Art Therapy and Artworks

Art therapy has long been recognized as a method that constitutes a primary process (Naumburg, 1950; Kramer, 1971; Levick, 1975; Wolf et al., 1977; Rubin, 1984) that taps into the non-verbal realm of imagery (Cohen and Riley, 2000; Talwar, 2007).

According to Gantt and Tinnin (2009), art therapists have responded with studies and reports on using art therapy with a wide range of trauma including disasters (Andruk, 1996; Howie et al., 2002), combating PTSD (Collie et al., 2006), childhood sexual abuse (Sweig, 2000; Murphy, 2001), adults (Waller, 1992; Rankin and Taucher, 2003; Spring, 2004), physical and medical trauma (Appleton, 2001; Chapman et al., 2001), treatment of dissociative disorders (Mills, 1995; Engle, 1997), and suggestions for education of art therapists who work with trauma survivors (Gonzales-Dolginko, 2003), among others.

Although there is much literature on the need to “make” art in therapeutic processes linked to trauma, there is a gap in studies on how “seeing” artistic works can help, in their potential for “intercommunication between the inner being of things and the inner being of the human self” (Maritain, 1953, p. 3).

Although there is still no consistent literature on artistic images, there is literature on the use of photography in therapy. Following Kwesell (2020), still photographs can participate in the trauma healing process through both the act of photographing or viewing still photographs because the eye has time to pause and the psyche time to process (Zelizer and Allan, 2011). Photo-elicitation interviewing (PEI) has been employed to unearth individual memories and emotions that are often hard to access in oral interviews (Collier, 1957), and photographing interventions such as auto-photography (Noland, 2006), photovoice (Wang and Burris, 1997), and photo interview (Kolb, 2008) have been used to examine identity, promote social change, and unearth community, socio-economic and environmental-level trends and concerns.

### Art Therapy, Systematic Reviews and Mixed Methods Research

In this same line of inquiry, in the pursuit of research methodologies that evidence the study of artistic images, it should be noted that systematic reviews or meta-analysis have been used in art therapy, and these are not directly oriented toward visual therapy resources. Far fewer have worked with mixed methods research (MMR) from a pragmatic paradigm.

Thus, Van Lith et al. (2013) analyzed primary studies that used quantitative, qualitative and mixed methods to address artistic creation and mental illness in adults through a critical systematic review. They found that practices based on art improved psychological comfort, specifically in the areas of self-discovery, self-expression, relationships and social identity. They also highlighted the need to work on the design of quality mixed methods that help integrate quantitative and qualitative research. In the years thereafter, Van Lith (2016) continued to study this topic by means of a systematic review, this time between 1994 and 2014, verifying that approaches in art therapy, when working with people with mental health problems, do not offer a description that enables transferability of this practice to other similar contexts, which would also help promote the development of a practice based on evidence in art therapy.

In a similar investigation, Abbing et al. (2018) centered their attention on systematic reviews of non-randomized controlled studies to evaluate the effects of art therapy on anxiety in university students, and explore the characteristics of the intervention and its benefits. They found that a variety of visual techniques, like trauma-related mandala design, collage making, free painting, clay work, still life drawing and house-tree-person drawing, promote the reduction of anxiety before examinations. Meanwhile, the narrative review of Wood et al. (2011) describes that the procedures mainly used in interventions with art therapy on patients with cancer are questionnaires, in-depth interviews, patients’ artwork, therapists’ narratives of sessions, and stress markers in salivary samples. Additionally, in the meta-analysis by Mi-Jin et al. (2016), clay showed to have great benefits in the psychological health of children, especially when applied in single or group sessions.

One of the conclusions of the narrative review of Attard and Larkin (2016) is the existing need to compare, contrast and integrate the quantitative and qualitative results in order to explore if therapy based on arts, applied to people with psychosis, is a meaningful intervention. They observed that there is discrepancy between the quantitative and the qualitative evidence. The first does not offer conclusive statistical results, while the second highlights the positive and meaningful value given in the feedback by therapists and patients.

Lastly, Archibald (2018) integrative systematic review is the only research that has explored the way in which arts and MMR seek to achieve a social benefit. This research, which synthesizes the findings in 26 primary studies, found that only six of them show integration between arts and MMR. This synergy is evidenced in the methodological processes or events examined for the development of a framework or intervention in art therapy. It concludes that it is necessary to head for an arts-MMR binomial that combines an artistic research methodology with an alternative paradigm based on its own methods.

In this theoretical and methodological context, this study is necessary because (a) there is evidence that esthetic images have motivating potential, (b) systematic studies about esthetic emotions and subjective changes are scarce, (c) exploratory research is intended to use art and the creation process in the treatment of trauma, (d) the few existing systematic reviews and meta-analysis in art therapy do not collect information about visual techniques, and (e) there is a need for there is a mixed research approach for the design and evaluation of a visual instrument for art therapy.

In this respect, the general objective of this paper is to design and validate the PROJECTA tool for therapeutic use, in order to address trauma or challenging situations in art therapy through artistic images and emotions, feelings or states of mind associated with those images. The specific objectives are (1) to analyze scientific articles related with the emotions that emerge in art therapy, (2) to select artistic images that allow intervention in therapy and art therapy, and (3) to evaluate the emotions, feelings or states of mind associated with those artistic images, as a useful orientation for health professionals.

The purpose of this research is to provide professionals with a resource in art therapy to assist in the identification, expression and demonstration of emotions or feelings related with trauma, or in challenging situations of vulnerability, psychological discomfort or post-traumatic stress.

## Methods, Materials, and Equipment

We used a mixed investigation approach, with collection, analysis and interpretation of qualitative and quantitative data to use both methodologies to increase the validity of the results obtained. It is a sequential concurrent design QUAL → quan, where qualitative research carries more weight than quantitative. Specifically, we used the concurrent triangulation design aiming to confirm, correlate or verify in the same study the results of a qualitative and quantitative integration (Johnson and Onwuegbuzie, 2004; Creswell, 2014).

**FIGURE 1:**
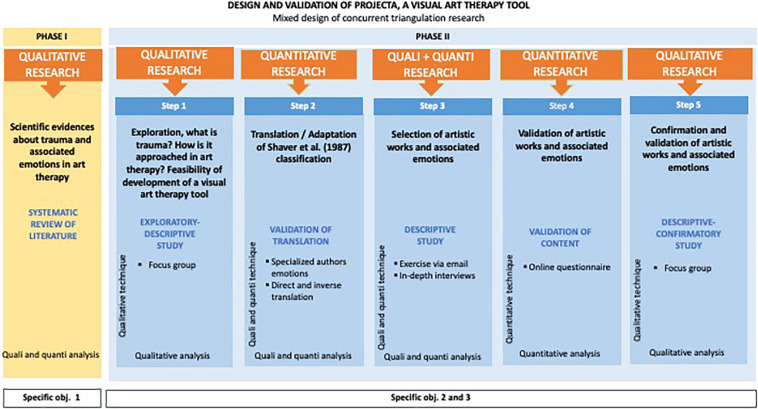
Mixed concurrent triangulation research design.

Figure 1 represents the *concurrent triangulation design*, a mixed research design for the development and validation of the visual instrument PROJECTA for art therapy.

### Procedure

#### First Phase

##### Systematic review

The objectives of this first phase were (a) to identify scientific documents that report emotions, feelings or states of mind in the process of art therapy, and, (b) to analyze in those documents the emotions that arise more frequently in art therapy, in situations of trauma or difficult conditions.

The *review of the literature* was carried out through the databases SCOPUS and Web of Science, with *descriptors* in English related with art therapy and professions linked with it (art therapy, artwork, therapy, psychotherapy, and professional), emotions, trauma, image (images, museum, photography, picture, visual art, and visual image), and context (abuse, cultural, gender, hospital, human trafficking, immigrants, mental health, violence, prostitutes, and refugee). These were combined and a search was performed in the title, keywords or abstract according to the requirements of each database.

The *inclusion criteria* were primary studies, (1) empirical or (2) or theoretical, (3) without timing criteria, (4) written in English or Spanish, (5) that include an abstract, (6) only research papers, (7) complete texts, and (8) related with art therapy in any area or context. The only *exclusion criterion* was if the art therapy had been conducted with children, teenagers, youths or older people (>75 years old), because the therapeutic instrument is intended for adults. People over the age of 75 years old were excluded because ages between 65 and 75 years old are still considered an early age of older adult (pre-old-age), while ages between 76 and 90 years old is considered advanced age. People over 90 years old are called “super-elders” (super-old-age) (Pérez and Abellán, 2018).

The *review of the primary studies process and its phases* was carried out in three rounds. In a *first round*, only 232 documents out of the 566 papers found met the inclusion criteria. Thus, 334 were discarded as they did not have an abstract (77), were duplicates (82), focused on children (86), teenagers (45), youths (22) and older people (12), or were about a book review (5), editorials (1), short communication (2), or dossier (2). The percentage of agreement among the judges was 89.76%.

In a *second round*, 155 of the 232 documents were discarded because they were not a complete text (154) or were written in a language other than Spanish or English (1). Consequently, only 77 met the inclusion criteria.

In a *third and last round*, all 77 documents were reviewed again in further detail. It was decided to also include the 155 documents with an abstract, although not all of them were complete texts, to include more information. The final sample consisted of 232 documents that met the inclusion criteria.

**FIGURE 2:**
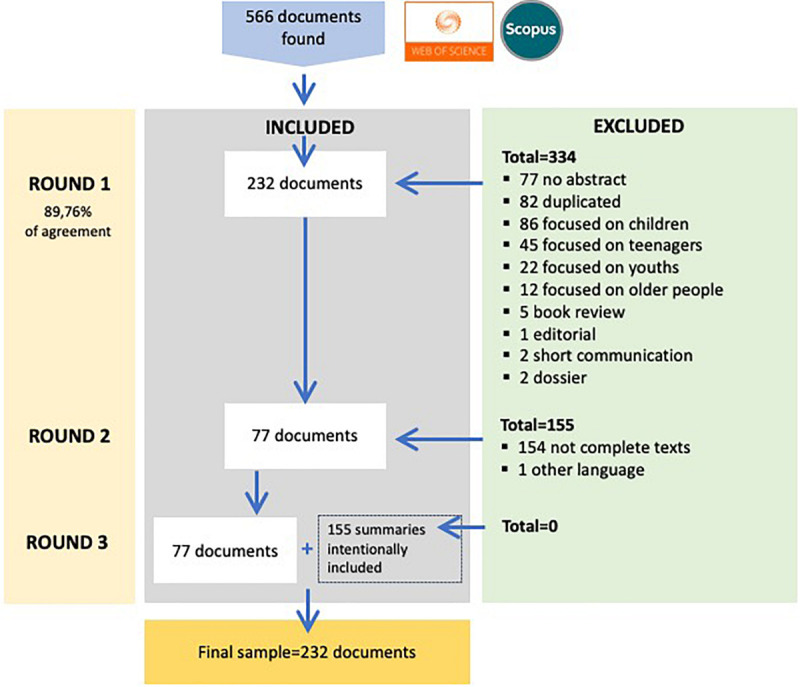
Flow chart.

Figure 2 represents a flow chart of the systematic review process and its phases, following the PRISMA statement (Preferred Reporting Items for Systematic Reviews and Meta-Analyses) (Hutton et al., 2016).

The *analysis of the final documents* included quantitative and qualitative techniques using Excel and Atlas.ti software version 8, respectively.

The qualitative analysis was achieved through two methodological strategies (Miles and Huberman, 1994). The first one was a content analysis following the guidelines provided by Erlingsson and Brysiewicz (2017) and working with the first four levels of abstraction: *meaning unit, condensed meaning units, code, category.* The fifth category, *theme*, was not used as it exceeded the objectives of this research phase. The second strategy used predetermined categories (positive and negative emotions from authors who were experts on the subject), by the use of the self-encoding function in Atlas.ti (strategy = expression, context = document, expansion = phrase). In the end, the descriptive statistics allowed us to qualitatively analyze the frequency and percentage of occurrence of these emotions.

#### Second Phase

##### Validation of images and emotions

The objectives of this second phase were: (a) to identify authors who were experts in emotions and their classification in the literature, (b) to select a proposal for the classification of emotions adapted to the field of art therapy, (c) to choose artistic works that facilitate working with art therapy, (d) to identify emotions associated with those artistic works and (e) to validate the predominant emotions linked to these artistic works that could be raised during the art therapy process.

*Step 1:* We established a first focus group consisting of 6 experts (1 art-therapist, 1 psychotherapist and art-therapist, 2 psychologists, 1 psychiatrist, and 1 psychoanalyst), in order to understand what trauma is and the way to approach it in art therapy, and, at the same time, to evaluate the feasibility for the development of a visual instrument for art therapy conducive to the identification of emotions.

*Step 2:* A review was conducted by *authors who were experts in emotions*, who mostly classify emotions as basic or positive and negative (Ekman et al., 1972; Lazarus, 1991; Lazarus and Lazarus, 1996; Fredrickson, 1998, 2001, 2009; Ekman, 1999; Bisquerra, 2009; Fernández-Abascal, 2009). The classification by Shaver et al. (1987) was chosen because it structures 146 emotions in a hierarchic abstract-concrete model based on six primary emotions. These are then broken down into 25 related secondary emotions and, from these, 115 tertiary ones. In this model, the broad scope of terms related with emotions, feelings or states of mind facilitated the means for searching the documents that were analyzed in the systematic review. Moreover, the authors themselves affirm that this extensive classification incorporates intercultural differences (Supplementary Material 1).

The proposal by Shaver et al. (1987) was translated and culturally adapted following the indications of Ramada-Rodilla et al. (2012), through a direct translation (from English to Spanish) in which two independent bilingual native Spanish speaking translators participated, and a reverse translation (from Spanish to English) by means of two independent bilingual native English speaking translators (Supplementary Material 2). The percentage of agreement was 99.5%.

*Step 3:* We invited 13 experts via email (4 art therapists; 1 art therapist and gender expert researcher; 1 drama therapist; 1 psychologist and researcher; 2 psychoanalysts and gender experts; 1 professor of history of art specialized in oriental and Hindu art; 1 professor of esthetics and art critic; 1 museum educator; and 1 artist and gender expert researcher) to perform the task that consisted of the selection of art treasures and association of them with any of the emotions in the Shaver et al. (1987) classification. Nine of the 16 experts also participated in in-depth interviews.

In the interviews, the experts justified why they had chosen the artworks and the association with that particular emotion and also reviewed Shaver’s proposal and adjusted it when they considered any emotion was missing. The experts suggested the incorporation of primary emotions like emptiness, victory, balance and sense of humor as these frequently emerge in art therapy when treating difficult or traumatic situations, either because they are present, excessive, or due a sense of longing, deficit or difficulty in feeling emotions.

Therefore, each expert had to search for at least 10 images, that is to say, one artwork for each one of the 10 primary emotions, but they also had to be related with their second or tertiary emotion (e.g., an image of fear as the primary emotion that could also represent horror or nervousness as a second emotion, or any of the associated tertiary emotions). There was also the possibility that they could choose a maximum of three images per primary emotion, for a total of 30 artistic treasures. There were four conditions for the selection of the images: (1) cultural diversity, (2) images of masculine or feminine authorship, (3) not only of European or North American origin, and (4) that allowed working with people who had faced difficult events and traumatic experiences and could help them think and delve into their own life experiences as well as rebuild their lives.

A total of 226 images were obtained. Out of that total, 19 were duplicates. The images associated with the same secondary emotion (6) were excluded; those that were duplicated but associated with a different secondary emotion were kept, leaving a total of 220 images.

*Step 4:* We developed a questionnaire in order to validate the art treasures and the associated emotions proposed by the 16 experts. The primary and secondary emotions chosen by the experts for each image were taken into consideration, ignoring the tertiary emotions in order to make the comprehension and response of the questionnaire quicker and easier. Therefore, images associated with a tertiary emotion were considered as the correspondent secondary emotion. The multiple choice questionnaire consisted of 220 images randomly distributed in 22 forms (each one with 10 images). Among the four possible answers to choose from, one of them corresponded to the emotion suggested by the expert, another suggested a very similar option, a third was completely unrelated to the emotion proposed by the expert, and the last one allowed the participants to write in their own choice from the complete list of emotions if they did not like the ones presented.

It was sent to 674 students and professionals in psychology, education, art therapy, fine arts, audiovisuals and film, obtaining 228 answers, which were quantitatively analyzed according to the percentage of agreement. The percentage of validation was established at 80% or more of coincidence in the association between artwork and emotion.

*Step 5:* Lastly, we invited six experts from a second focus group (2 gender expert psychoanalysts, 1 art therapist and gender expert researcher, 1 doctor in art therapy, 1 doctor in education, and 1 psychologist and art therapist) to participate in the validation of the results obtained from the online questionnaire for two sets: the images that obtained more than 80% agreement, and the images with less than 80% agreement, but that are considered equally interesting for the instrument or to search for new images for emotions like victory, due to the variety of the answers obtained. Its aim was also to propose new artworks for emotions considered interesting and necessary for the instrument like guilt, shame, self-compassion and restlessness, all of them frequently exposed in a trauma state.

Additionally, we added up a last group of images called ambiguous or stimulating, consisting of five images that were initially proposed by the experts, but obtained a very low percentage of agreement in the questionnaire answers and did not obtain a majority consensus in the same focus group. It was agreed to include them in the instrument due precisely to the polysemy of meanings that they aroused.

In this second phase, the quantitative analysis was used for the analysis of both focal groups, in-depth interviews, and the review of authors who were experts in emotions. The qualitative analysis was used to obtain the percentage of agreement for the validation of the translation of the proposal by Shaver et al. (1987), the answers obtained for the artwork-emotion association, and the percentage of agreement in the answers to the online questionnaire.

## Results

### Systematic Review

**FIGURE 3:**
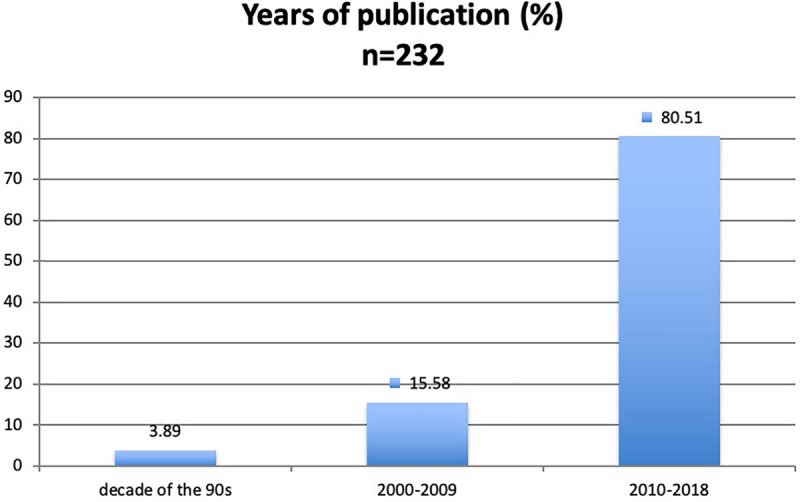
Percentage of articles by year of publication.

In the 232 documents that met the inclusion criteria, it was observed that most of them (80.51%) were published between 2010/2018, followed, not so closely, with 15.58% that were published between 2000 and 2009. The decade of the 90s had the lowest percentage of publication (3.89%) (Figure 3).

That said, when the documents were qualitatively analyzed, 6,561 references reported emotions. More than half of those references, 56.90% (*n* = 3733) referred to negative emotions, feelings and states, while to a lesser extent, 43.10% (*n* = 2828), were associated with positive emotions, feelings and states. It was decided to analyze positive and negative emotions, but not mixed, because this is the classification more frequently used in scientific literature, criterion that favored the search for documents (Ekman et al., 1972; Lazarus, 1991; Lazarus and Lazarus, 1996; Fredrickson, 1998, 2001, 2009; Ekman, 1999; Bisquerra, 2009; Fernández-Abascal, 2009). The references were analyzed again in order to regroup the emotions obtaining 157 emotions, 89 of which were classified as negative (56.69%) and 68 as positive (43.41%) (Table 1).

**TABLE 1:** Frequency and percentage of quotes and emotions.

**Emotions**	**No. of references**	**% of references**	**No. of emotions**	**% of emotions**
Negative	3733	56,90	89	56,69
Positive	2828	43,10	68	43,41
Total	6561	100	157	100

A more detailed analysis of these emotions indicated that the 10 negative primary emotions that emerge more frequently in the therapeutic process referred to depression (27.16%), anxiety (24.51%), anger (5.19%), sadness (4.28%), fear (3.93%), exclusion (3.05%), shame (2.97%), loneliness (1.87%), guilt (1.74%) and discomfort (1.58%). The 10 positive primary emotions referred to positivity (21.67%), understanding (10.53%), present (9.90%), interest (4.31%), expressiveness (3.74%), focused (3.43%), courage (3.11%), happiness (3%), active (2.65%), and bond (2.61%) (Table 2).

**TABLE 2:** Frequency and percentage of emotions, feelings and states of mind.

	**Emotions, feelings, and states of mind**	**N°**	**%**
Negative	(1) Depression	1014	27,16
	(2) Anxiety/uneasiness	915	24,51
	(3) Anger/angry/	194	5,19
	(4) Sadness/sorrow/gloom/melancholy/longing	160	4,28
	(5) Fear/apprehension/afraid/alarm/	147	3,93
	(6) Excluded	114	3,05
	(7) Ashamed/embarrassed	111	2,97
	(8) Loneliness/desolation/isolation	70	1,87
	(9) Guilt/remorse	65	1,74
	(10) Discomfort	59	1,58
Positive	(1) Positivity/positive/wellness	613	21,67
	(2) Understanding	298	10,53
	(3) Present	280	9,90
	(4) Interest	122	4,31
	(5) Expressiveness	106	3,74
	(6) Focused	97	3,43
	(7) Strong/courage	88	3,11
	(8) Happy/happiness	85	3,00
	(9) Active	75	2,65
	(10) Connection/bond	74	2,61

At the same time, the 157 emotions found in the literature were compared with the proposal by Shaver et al. (1987), with a 74.55% match in negative emotions, while the percentage of match in positive emotions was much lower, only 36.4%.

### Validation of Images and Emotions

As a result of the qualitative analysis from the first focus group, three major categories emerged: trauma, emotions and benefits of working with the image.

The understanding of trauma was a wide-ranging debate due to the conceptual and methodological complexity of the construct. For some experts, trauma is an experience that we all pass through, a specific event or a threat to physical life that blocks development. This blockade causes an individual to live an unfulfilled life, and sometimes the person forms their character around that traumatic experience. Others who have worked with vulnerable minors refine the concept and define it as the persistent lack of attachment figures, and the way this interferes in the development of the minor. However, everyone agrees that each case is unique and complex in itself. Some people are able to process the trauma while others lack resources to do so. Those who have more personal resources process it better and the traumatic part “*is that excess.”*

One of the experts, who has coordinated programs with individuals with AIDS and HIV, states that, after being diagnosed, these people experiment deep changes in life style and the feelings that emerge more frequently are guilt and anger, which emerge in an abstract and undetermined way. He also states that people who have suffered trauma tend to experiment a burden, a personal guilt. The lack of emotional expressiveness and the inability to connect are present in those who have experienced a trauma.

Some experts who work with photo-elicitation with the general population, but also with people with traumas, have experienced the way this tool helps emotions to emerge. This is a resource that allows the person to look at the content of the trauma indirectly because, on many occasions, it would not be possible to do it directly. Another expert uses short films to discuss with his patients and agrees with those who state that images give individuals without resources the opportunity to understand their traumas and start communicating and expressing themselves. The patient begins to communicate trauma when an opening is produced as a consequence of a safe and confident space in the therapeutic environment.

Artworks are means, resources, tools that facilitate the understanding of trauma, because sometimes the person *“does not even have anything to deliver anywhere.”* Their power has not been fully explored and their therapeutic use shares ethical limits with the discipline itself. Three experts pointed out the importance of respect and accepting each individual’s paces, so that the images do not force processes that the person is not able to close, or simply does not want to open. Empathy, time and knowledge are some of the key elements in the therapeutic process. All of the experts believe that working with artworks associated with emotions is necessary, as a contributing factor for art-based therapy.

**FIGURE 4:**
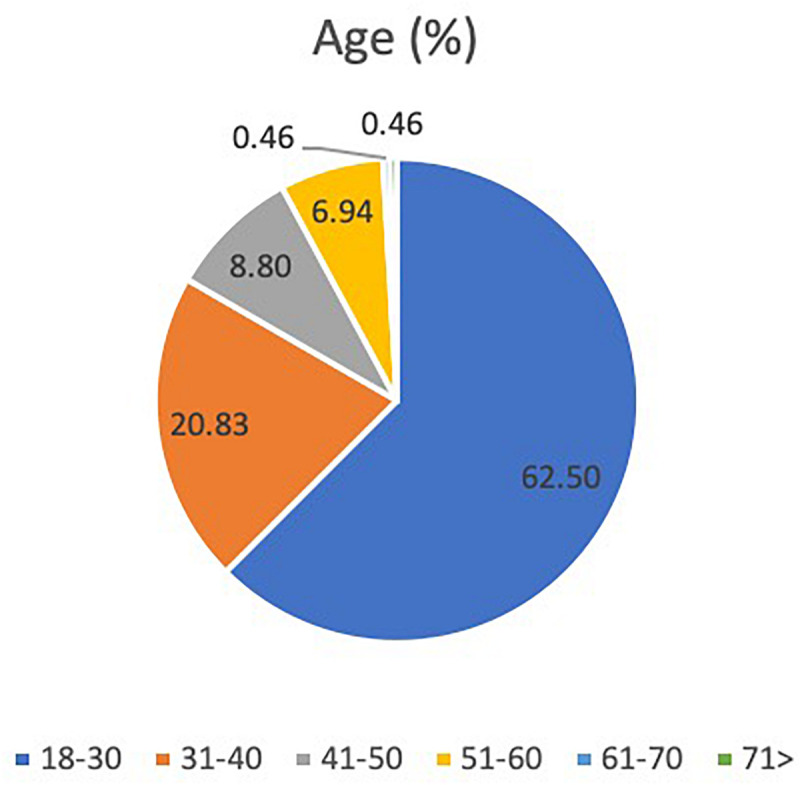
Percentage of questionnaire participants by age.

Regarding the results of the online questionnaire, out of the 228 subjects that participated, 83.3% were female and 16.67% male, with ages ranging from 18 to 75 years old (Mean = 31 and DT = 10.66). More than half of the sample (83.3%) was between 18–40 years old, 62.5% between 18–30 years old and 20.83% between 31–40 years old. The remaining 16.67% was divided into 8.80% who were between 41–50 years old, 6.94% between 51–60 years old, 0.46% between 61–70 years old and 0.46% over 71 years old (Figure 4).

**FIGURE 5:**
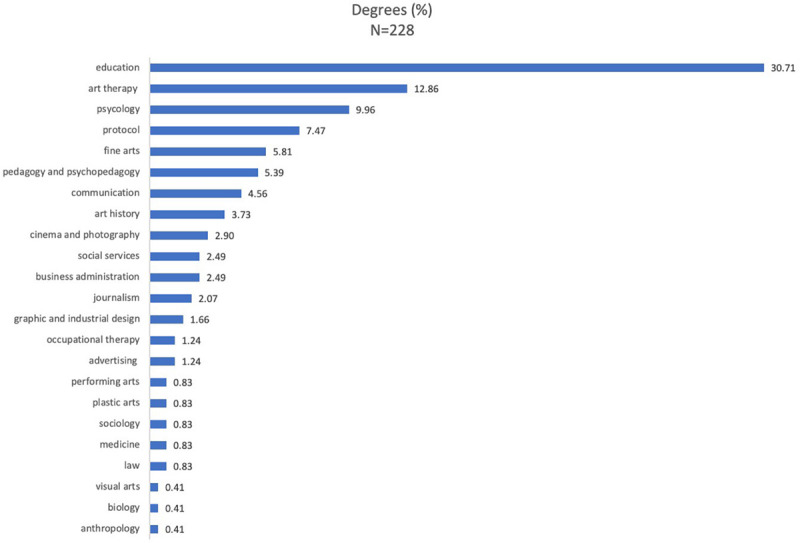
Percentage of questionnaire participants by career degree.

Most of them came from degree programs in Education specializing in childhood, primary and social education (30.71%), and in arts (27.39%), with specializations in art therapy (12.86%), fine arts (5.81%), history of art (3.73%), film and photography (2.90%), and plastic, performing and visual arts (2.07%). Other professional qualifications with lower percentages were psychology (9.96%), communication, journalism and publicity (7.88%), protocol and event organization (7.47%), and social work (2.49%) (Figure 5).

Only 12.96% of the participants have no work experience, while 87.04% have professional experience. Out of these, 75.46 have work experience between 1 and 15 years, 7.41% between 16 and 30 years, and 4.17% of more than 30 years.

**FIGURE 6:**
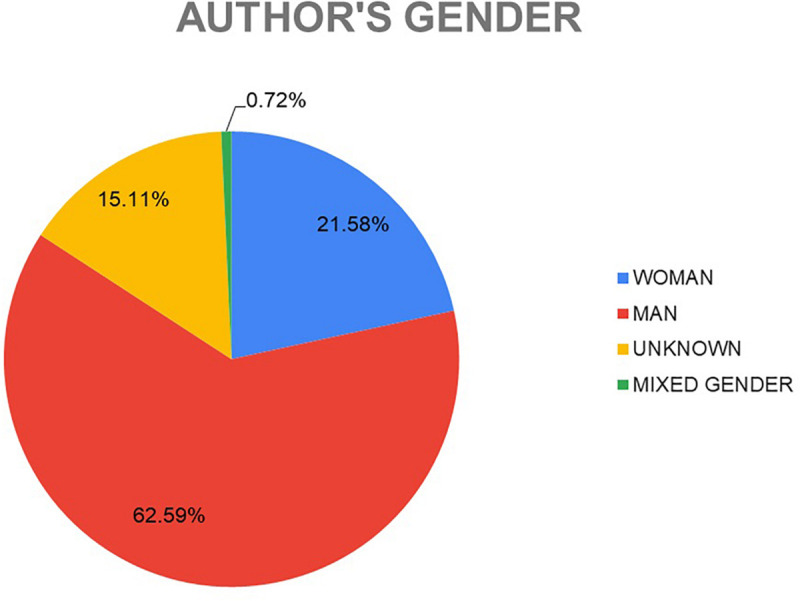
Author’s Gender of the works selected by the experts.

Concerning the images selected by the experts, taking into account the gender of the authors of the works, it has been observed that 62.59% of them belong to male authors, and 21.58% to women; 0.7% are made up of artistic groups with members of both genders and for the remaining 15.11% the gender of the authors is unknown (Figure 6).

**FIGURE 7:**
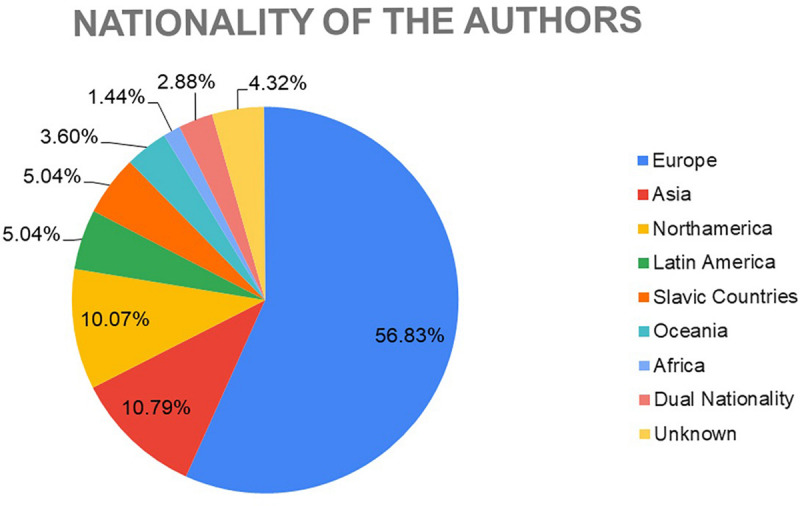
Author’s nationality of the works selected by the experts.

With regard to the origin of the authors of the works selected by the experts, the highest percentage was found to be of European origin (56.83%), followed by Asia (10.79%), North America (10.07%), Latin America (5.04%), Slavic countries (5.04%), and Oceania (3.60%). It has also been found that 2.88% of the selected authors have dual nationality, and finally, there are 4.32% of anonymous authors and/or of unknown origin (Figure 7).

Once the expected number of responses to the questionnaires was obtained, they were analyzed with the aim of observing which images achieved a high percentage of agreement with the emotion chosen by the expert and which did not reach that percentage. A high rate of consensus was considered to be reached if there was 80% or more agreement between the emotion chosen by the expert and the answers obtained on the forms.

It was assumed that the answer was consistent with the expert’s choice in the following cases: (1) if the person had chosen the option with the emotion chosen by the expert, (2) if he or she had chosen the option with the emotion very similar/very close to the one chosen by the expert or (3) if he or she had chosen the free response option, but the emotion chosen was still very similar/very close to the one initially chosen by the expert for that artwork.

They were considered not similar if (1) the person had chosen the option with an emotion totally opposite to the one chosen by the expert or (2) the person had chosen the free response option and the suggested emotion was very far/totally opposite to the one chosen by the expert.

**FIGURE 8:**
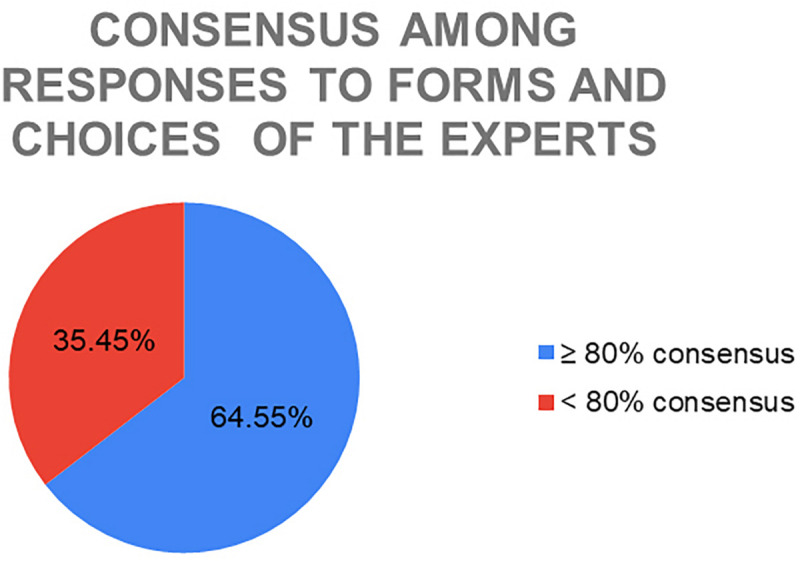
Consensus among responses to form and choices of the experts.

Thus, observing the answers obtained by each of these 220 images, it was found that 64.55% of the images (142) have achieved 80% or more similarity between the emotion chosen by the expert and the emotion selected in the questionnaires, while 35.45% of the remaining works (78) obtained a consensus of less than 80% (Figure 8).

**FIGURE 9:**
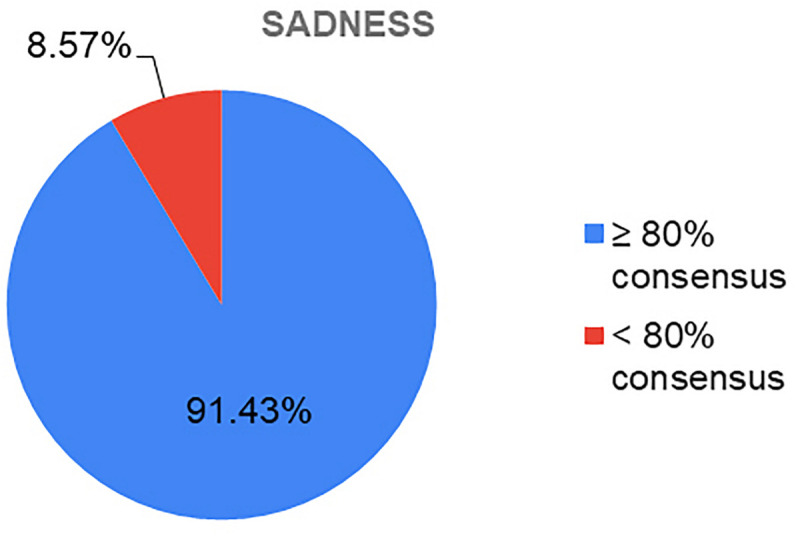
Consensus percentages obtained for the emotions of sadness.

Looking at the degree of consensus that each emotion has obtained, it can be seen that *Sadness* (Figure 9) is the primary emotion with the highest percentage of consensus (91.43%). This may be due to the fact that *Sadness* is a more easily identifiable emotion, as it is associated with a more recognizable symbolic language and has been more commonly represented in culture throughout history. In addition, as discussed above, negative emotions tend to predominate in therapy, as well as in the scientific literature devoted to the study of emotions.

**FIGURE 10:**
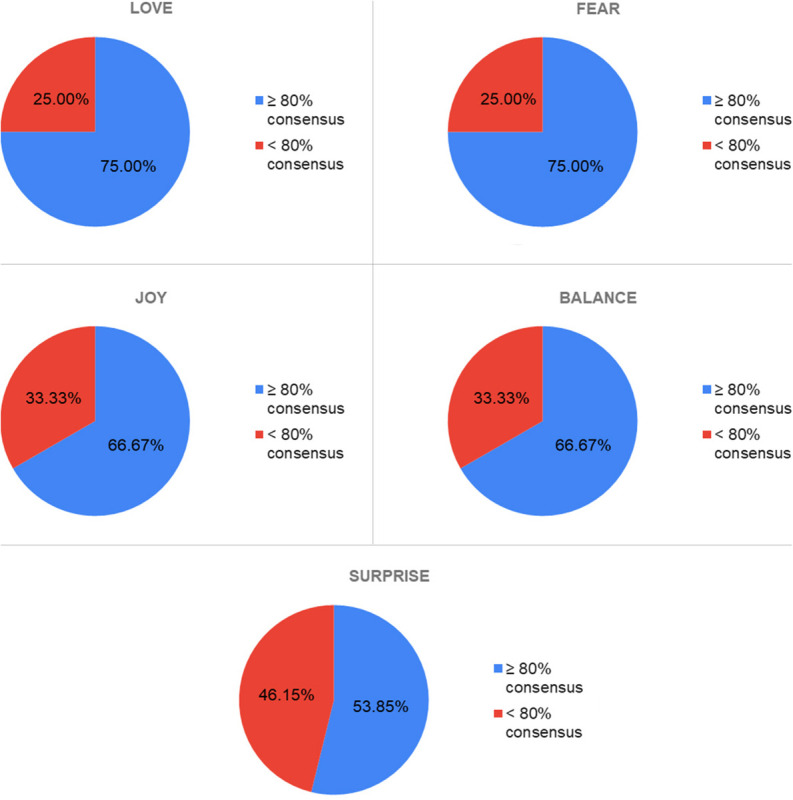
Consensus percentages obtained for the emotions of love, fear, joy, balance, and surprise.

Other primary emotions (Figure 10) such as *Love* and *Fear* have achieved a degree of consensus of 75%, which although lower than in the case of *Sadness*, is higher than in the case of *Joy* and *Balance* (66.67%) or *Surprise* (53.85%), perhaps, as was the case with *Sadness*, because these two emotions are more easily identifiable and have been frequently represented throughout the history of art. However, *Surprise*, on the other hand, may be a more ambiguous emotion and more difficult to identify in a univocal way, and is also an emotion of transit to another (fear, joy, humor, and anger).

**FIGURE 11:**
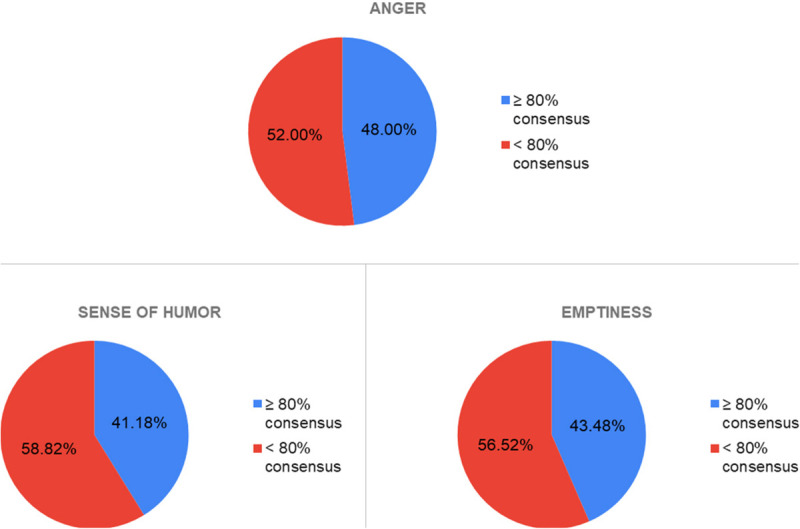
Consensus percentages obtained for the emotions of anger, sense of humor and emptiness.

Finally, emotions such as *Anger* (48% consensus), *Emptiness* (43.48%), and *Humor* (41.18%) have achieved a much lower percentage of agreement than previous emotions. In the case of *Emptiness* it may be because it is a more complex and specific emotion in traumatic situations (it should be remembered that this emotion was added from the first focus group); it is also an emotion related to culture. In the case of *Humor*, it may be because it is an emotion closely linked to background, culture and one’s own life experience. Finally, with respect to *Anger*, it is an emotion that can share characteristics with *Fear* or *Sadness*, which can make it difficult to recognize and represent (Figure 11).

Once the results of the online questionnaires were analyzed, a second focus group was called in order to confirm the results obtained.

First, the emotions that were finally most relevant to the tool were chosen (Supplementary Material 3). Among them, the emotion of self-compassion was proposed, which had not been reflected until now in the table of emotions, but which was considered relevant in the treatment of trauma. Filial love and conjugal love were also proposed as secondary emotions in Love, to qualify the type of attachment.

The experts were then asked to review the images that had obtained less than 80% consensus in the questionnaires and that were related to the emotions chosen in the previous step, in order to check whether, despite not having obtained a wide consensus on that emotion, they could be interesting in the new tool. The images with less than 80% consensus that were finally selected for their relevance to the final tool were: The Temple of Juno in Agrigento, Caspar David Friedrich (linked to Love: Nostalgia, 60% consensus); Market at Minho, Sonia Delaunay (linked to Alegria, 60% consensus); The Return of the Pigeon to the Ark, J. E. Millais (linked to Alegria: Hope, 40% consensus); Self-Portrait, Käthe Kollwitz (linked to Equilibrium, 40% consensus); Peaceful Fishing in a River in the Autumn, Ink on Paper, Song Dynasty of the South, Ma Yuan (linked to Equilibrium: Calm, 71.42% consensus); Circus 2, Gaston Izaguirre (linked to Anger: Rage, 66.67% consensus); Hotel Room, Edward Hopper (linked to Sadness: Solitude, 70% consensus); Florentine Pietà, Michelangelo (Love: Affection; 57.14% consensus); The Arch of Hysteria, Louise Bourgeois (Fear: Horror; 70% consensus); The Persistence of Memory, Salvador Dalí (Fear: Unrest; 66.66% consensus); Ofelia, John Everett Millais (Emptiness; 60% consensus); Young Slave, Michelangelo (Emptiness: Incapable; 66.66% consensus); Red Hysteria, Marina Núñez (Emptiness: Disconnection; 70% consensus); The Melancholy and Mystery of a Street, Giorgio de Chirico (Emptiness: Disconnection; 28.57% consensus).

In this review process it was also agreed that none of the images associated with the emotion *Victory* were sufficiently representative of this state, and furthermore, it was proposed to change the term because it was considered to resonate too much with sport, with competition, and in its place the concept of *Overcoming* was proposed. Accordingly, the following images were proposed: Freedom guiding the people, Eugène Delacroix; Le Petit Prince, Lita Cabellut; Helreiðin (staekkun); Ásmundur Sveinsson.

It was also proposed to change the term *Emptiness* as it is difficult to understand, to *Emptying*, and *Disconnection* to *Disconnected*.

Emotions or feelings such as Guilt, Restlessness and Shame were established as important in the final tool, as they often emerge in the therapeutic process, but in the first selection of experts they had 2 or less associated images, as they were sub-emotions in many cases linked to culture and the socialization process. In addition, as mentioned above, self-compassion was included, as it is a key emotion for the processes of overcoming trauma. Between five and eight images were proposed for each one and those finally chosen were: Dreams of Fatigue, Grete Stern (Guilt); Woman Thinking, Käthe Kollwitz (Shame); Fear (Das Bangen), Käthe Kollwitz (Self-compassion), and Little Night Music, Dorothea Tanning (Restlessness).

There were also several images that, although initially linked to an emotion, focus group experts considered that they better represented others: No title, no. 4. Abortion series, Paula Rego (initially linked to Shame, finally linked to Abandonment); Dreams of the Mirror, Grete Stern (initially linked to Restlessness, finally proposed for Horror) and India (Punjab Hills, Kangra) (c.1800). The Timid Bride (initially linked to Emptiness: Diminished/small: Shyness, finally linked to Anger: Disgust).

Finally, it was decided to collect in a new group those images that had not obtained enough consensus in relation to the emotion chosen by the expert for that work, neither in the questionnaires nor in the focus group, but were considered to be very mobilizing images and that could open a polysemy of interesting emotions for intervention in people with traumas. These images were: The comforter, Patricia Piccinini (initially proposed for Love: Affection: Sensitivity), Death and life, Gustav Klimt (initially proposed for Fear: Horror); Personen, Miriam Cahn (initially proposed for Guilt); L’homme qui marche, Giacometti (initially proposed for Emptiness: Diminished/small: Incapable); Inquisition, Edouard Moyse (initially proposed for Fear: Horror).

A final list of 92 images was obtained, related to the emotions chosen for the PROJECTA tool (Supplementary Material 4).

## Discussion

In this process, the artistic image has demonstrated its potential to simultaneously summon a memory crossed by cognitive and emotional elements. Aesthetic contemplation moves to a special state of existential apprehension similar to the space of therapy, by attracting past experiences to the present moment. As a symbol, the artistic object activates vital states by showing alternative visions of the relationship between the self and the world. The process carried out through long interviews with experts, and then validated through the questionnaire and the focus group, shows the evocative power of artistic images to convoke a new critical consciousness in a safe space through aesthetic appropriation, without the painful effects that a past experience could have. Crossed by culture, socialization and the person’s biography itself, the polysemy of artistic work is shown as an enriching and essential element where each human being finds a vital mirror in which to reflect him or herself. A place where there are no closed answers, and where alternative visions can be discovered of her or his own existence and, most importantly, confirmation of the value of his or her suffering and struggle to survive.

On the other hand, emotions are a complex and multidimensional construct in which social, cognitive, biological and cultural aspects intervene in a direct or indirect way (Matsumoto et al., 2008), and that (a) have a precise and subjective connotation or meaning for each individual, (b) are accompanied by a particular and very specific facial expression, (c) have a specific pattern of neuronal discharge, in other words, a single and particular physical reactions, and (d) generate particular behaviors that can be used to recognize each specific one (Maganto and Maganto, 2013).

Despite the conceptual and methodological challenge assumed by addressing this complexity, emotions have been and still are studied mainly by Psychology on a long journey where experts in the field continue to work to understand how negative and positive emotions coexist, complement each other, and play a part in human nature (Fredrickson, 2002; Carr, 2007).

However, there is a trend that can be seen in the scientific literature and the primary studies of the systematic review that was carried out: negative emotions have been more thoroughly studied than positive ones. This does not mean that Shaver’s approach is not suitable, but that, in general, scientific papers tend to report a greater number of negative than positive emotions during therapeutic processes, a fact that confirms, once again, the results in Table 1. The reason for this is that negative emotions have an adaptative function, offer clear and specific answers, are tendencies for action and add substantial value in the immediate survival of the human being. In contrast, positive emotions are harder to identify and define. Positive is assumed as part of what is expected and answers are not as clear and specific. Moreover, research on pathological problems has generally left behind the investigation of the positive and, thereby, the strengths, virtues and potentials of the individual.

Precisely, this prevalence of negative emotions in the scientific literature can be related to the results obtained regarding the percentage of consensus generated by the emotion of Sadness, since in this case it is the primary emotion that has achieved a higher percentage of consensus, as well as that for which more artistic images were received.

As mentioned at the beginning of this section, emotions are crossed by cultural differences, the process of socialization and the uniqueness of each subject, understanding the vital experiences of each one by their perception, which is more perceptible in complex emotional states. In this sense, results show that emotions such as Emptiness have obtained a lower percentage of consensus than others such as Love, which may be due to the fact that it is a complex emotion whose meaning varies radically in relation to the point of view from which it is considered. On the one hand, it is a very frequent state in PTSD and other traumatic processes and present in the dissociative symptoms of de-realization and depersonalization, as defense and adaptation mechanisms in the face of overwhelming situations (Van der Kolk, 1994). On the other hand, in cultures/philosophies, religions and other spiritual conceptions, as is the case of Buddhism, emptiness/vacuity -known as Shuni- is a polysemic concept that refers to the state of meditation, the deep understanding of reality and the no-self or lack of ego. In this case, emptiness would be a positive state to which to aspire (Arnau, 2005). Other emotions like Love and Fear have achieved the highest percentage of consensus in our results, maybe because these emotions are more easily identifiable and have been frequently represented throughout the history of art.

Emotional states such as guilt and shame are also strongly nuanced by culture and gender-differentiated socialization (Efthim et al., 2001; Silfver, 2007; Furukawa et al., 2012), so presumably the number of images obtained for these emotions was very low. In the case of sense of humor, it also seems to be influenced by cultural and generational differences (Olsson et al., 2001; Martin, 2007), gender (Wu et al., 2016), individual aspects through various cognitive processes, motivation and previous affective implications (Kuiper et al., 1995) and contextual factors (Booth−Butterfield and Booth−Butterfield, 1991). Hence, the consensus obtained for this emotion was lower than in other more immediate emotions related to the survival mechanisms of the individual, as may be the case of Fear.

Finally, Self-compassion and Overcoming were two states that were later included as a result of what was reported in the focus groups and the in-depth interviews with the specialists in trauma therapies, since they are two psychological constructs that help and favor the therapeutic process: on the one hand, overcoming as a desire and final objective, and self-compassion as a possible auxiliary method. Various researches show that self-compassion is a factor related to psychological well-being, mental health and prevention of psychopathology (Thompson and Waltz, 2008). Furthermore, it has been shown that interventions based on self-compassion decrease the severity of symptoms related to avoidance (Seligowski et al., 2014) and they can thus improve the ability to support and re-historize traumatic memory the possibility that the person can be exposed to these traumatic memories in a safe manner (Germer and Neff, 2015).

The challenge for professionals linked to art therapy is to keep up with methodologies and observation-evaluation strategies that develop emotions, feelings and states of mind, as a source of storage of psychological, emotional and social resources, in order to promote adaptation, enlargement of thought and action, environmental exploration, personal growth and, especially, the ability to address future difficult circumstances.

The identification and expression of emotions offers health benefits, subjective well-being and resilience (Fredrickson, 2002), creativity, openness to new experiences, acceptance of critical feedback, and the experience of a “unity with the others” (Garland et al., 2010), all reasons that support the development of visual art therapy tools such as PROJECTA.

Last but not least, the methodology used in the present investigation adds additional value, because very few studies include mixed methods research for the validation of visual art therapy tools. Authors like Gerber (2016) affirm that it is necessary to stop, identify and select the most suitable, systematic, and rigorous methodologies in favor of an investigation that contributes to the nature and vision of art therapy. That is why “mixed methods research provides viable options for advancing the art therapy research agenda while preserving the essential meaning of the field” (p. 654). Archibald and Gerber (2018) also claim that the integration of arts with mixed methods research has an unexplored potential, because both perspectives seek to understand social and cultural complexity for a transformative change through innovative methodological approaches. Mixed designs are congruent with the forms of knowledge of art therapy and promise progress in this field with philosophical and methodological integrity (Betts and Deaver, 2019).

In fact, methodological pluralism provides a critical and pragmatic response to complex ever-changing social problems using multiple methods to address an issue, which also depend on epistemological pluralism, understood as the recognition of the legitimacy of the sources and types of knowledge (Popa and Guillermin, 2017).

## Conclusion

Art has been a necessary vehicle in all cultures to share states of attachment, separation or loss. Authors such as the anthropologist Ellen Dissanayake (1988, 1992) point to Art, play and ritual, as essential activities for the psychic survival of human groups. In their need to build objects with a symbolic charge that would help them to understand themselves and endure, human beings made art a transitional space where they could understand themselves in the world. This space offered them skills of symbolic adaptation through the development of a holistic, transcendent understanding, united with the ability to imagine an alternative present. The images and objects of Art present in museums, from different periods, geographies and cultures, have served, both now and in the past, to establish an encounter with what is possible, beyond the contingencies of humans. Art, in its in-seeing potential, brings together intellectual, affective and unconscious identification, revealing itself as an indispensable instrument to help one understand and structure one’s own suffering.

Cultural spaces, which display works of art, and therapeutic spaces where professionals can accompany people who have suffered the psychological consequences of traumatic events, are emerging as places with immense potential where aesthetic perception can help to see “further in and beyond.” This work has revealed the importance of furthering the studies on contemplation and aesthetic perception initiated in the 19th century in relation to empathy and therapeutic potential and the great field of cultural spaces today that are sued not only for the recognition of heritage, but as symbolic spaces of personal development. As Coles (2020) suggests, museums-based Art therapy can articulate key therapeutic principles of holding, containing, trust, exploration and learning, play and acceptance that can be used by Art Therapists within their practice.

PROJECTA has been created to allow the identification, expression and demonstration of emotions by means of artworks during therapeutic intervention, thus it does not pretend to be a diagnostic or projective tool. Aware of the difficulty to establish associations between images-emotions, it does not seek to reduce the image to a single and obvious meaning (monosemic image), but expects the interpretation to be mediatized by those who observe, according to their personality, previous experiences, social relationships, culture… (polysemic image).

The instrument and its methodological process are not exempt from limitations. Since it is an exploratory study with a reduced sample, concordance coefficients have not been utilized to obtain significant differences between images and the associated emotion. The sample that responded to the questionnaire is also not representative. To compensate for the deficiencies, the triangulation of different sources and techniques has been used throughout the study. Despite this, a balance between images from masculine and feminine authors has not been achieved. Consequently, in other versions of the instrument this condition has to be considered.

It is expected that the implementation of the tool in future investigations will meet this broad and inclusive criteria. Another conceptual and methodological challenge will be to obtain a more effective reduced version of the images to work with people with traumas, through the validation by practitioners or in spaces like museums. The aim is also to elaborate a version directed at children who have had trauma or experienced difficulties. For these cases, artworks have to be in accordance with their evolutionary stage, social environment and cultural diversity.

It is recommended that the approach by Shaver et al. (1987), which was adapted with input by experts using a primary, secondary and tertiary emotion model, be continuously refined for its use in observation, intervention and evaluation methodologies.

Finally, investigations in art therapy with mixed methods research should consider the transformative and critical possibilities of arts. It is important to look at how these could be positioned for the integration of arts-MMR as a binomial, and not as a praxis where arts are embedded in MMR, to create instead a therapy based on art and a research methodology working together to push its own limits (Archibald, 2018).

## Data Availability Statement

The original contributions generated for this study are included in the article/Supplementary Material, further inquiries can be directed to the corresponding author.

## Ethics Statement

The studies involving human participants were reviewed and approved by University’s Faculty Research Ethics Committee. The patients/participants provided their written informed consent to participate in this study.

## Author Contributions

CC-T: elaboration of the phases for the mixed design of concurrent triangulation in order to integrate quantitative and qualitative results, analysis of the systematic review results, analysis of the general characteristics of the subjects that responded the online questionnaire and focus group 1, and application of the Atlas.ti program. MF-C: theoretical foundation of studies on trauma, its systematization in the field of psychology and its therapy in recent decades, theoretical foundation related to the aesthetic perception associated with the contemplation of works of art, their empathic potential and the possible psychoanalytic and therapeutic projection of the works of art in a therapeutic setting, and contributions of art therapy in situations of trauma and the use of images in therapy, and selection of experts, designed, and coordination the in-depth interviews. LF-E: collection and organization of the images chosen by the experts, with their corresponding emotions, quantitative analysis of the images chosen by the experts regarding the gender and origin of the authors, elaboration of the questionnaires (Google Forms), analysis of the responses to the questionnaires and focus group 2, and preparation of the final list of images and emotions from the analysis of the results of the questionnaires and focus group 2. All authors contributed to the article and approved the submitted version.

## Conflict of Interest

The authors declare that the research was conducted in the absence of any commercial or financial relationships that could be construed as a potential conflict of interest.
