# Supplementary material for: PROJECTA: An Art-Based Tool in Trauma Treatment
Source: Front Psychol. 2020 Dec 18;11:568948. doi: 10.3389/fpsyg.2020.568948 (PMC7775401; doi:10.3389/fpsyg.2020.568948)
Supplement: Supplementary file 1 [file Table_1.docx]

**Supplementary Material 1**

Classification of emotions according to Shaver et al. (1987)

| **Primary emotion** | **Secondary emotion** | **Tertiary emotions** |
| --- | --- | --- |
| Love | Affection | adoration, fondness, liking, attraction, caring, tenderness, compassion, sentimentality |
|  | Lust/sexual desire | desire, passion, infatuation |
|  | Longing | longing |
| Joy | Cheerfulness | amusement, bliss, gaiety, glee, jolliness, joviality, joy, delight, enjoyment, gladness, happiness, jubilation, elation, satisfaction, ecstasy, euphoria |
|  | Zest | enthusiasm, zeal, excitement, thrill, exhilaration |
|  | Contentment | pleasure |
|  | Pride | triumph |
|  | Optimism | eagerness, hope |
|  | Enthrallment | enthrallment, rapture |
|  | Relief | relief |
| Surprise | Surprise | amazement, astonishment |
| Anger | Irritability | aggravation, agitation, annoyance, grouchy, grumpy, crosspatch |
|  | Exasperation | frustration |
|  | Rage | anger, outrage, fury, wrath, hostility, ferocity, bitterness, hatred, scorn, spite, vengefulness, dislike, resentment |
|  | Disgust | revulsion, contempt, loathing |
|  | Envy | jealousy |
|  | Torment | torment |
| Sadness | Suffering | agony, anguish, hurt |
|  | Sadness | depression, despair, gloom, glumness, unhappiness, grief, sorrow, woe, misery, melancholy |
|  | Disappointment | dismay, displeasure |
|  | Shame | guilt, regret, remorse |
|  | Neglect | alienation, defeatism, dejection, embarrassment, homesickness, humiliation, insecurity, insult, isolation, loneliness, rejection |
|  | Sympathy | pity, mono no aware, sympathy |
| Fear | Horror | alarm, shock, fear, fright, horror, terror, panic, hysteria, mortification |
|  | Nervousness | anxiety, suspens, uneasiness, apprehension (fear), worry, distress, dread |
