# Supplementary material for: PROJECTA: An Art-Based Tool in Trauma Treatment
Source: Front Psychol. 2020 Dec 18;11:568948. doi: 10.3389/fpsyg.2020.568948 (PMC7775401; doi:10.3389/fpsyg.2020.568948)
Supplement: Supplementary file 2 [file Table_2.docx]

**Supplementary Material 2**

Translation and adaptation of the classification of emotions proposed by Shaver et al (1984)

| **Emociones primarias** | **Emociones secundarias** | **Emociones terciarias** |
| --- | --- | --- |
| Amor | Afecto | admiración, adoración, afabilidad, agrado, amabilidad, atracción, cariño, sensibilidad, ternura |
|  | Deseo sexual | deseo, ganas, impulso, lujuria, pasión, placer/disfrute sexual, seducción sexual |
|  | Nostalgia | añoranza, anhelo, deseo, morriña |
| Alegría | Entusiasmo, alboroto | dicha, disfrute, diversión, euforia, felicidad, gozo, jovialidad, júbilo, regocijo |
|  | Placer, diversión | agitamiento, emoción, excitación, fervor |
|  | Contento | placer |
|  | Optimismo | esperanza |
|  | Encanto | éxtasis |
|  | Alivio | alivio, levedad |
| Victoria | Satisfacción, triunfo, orgullo |  |
| Sorpresa | Sorpresa | asombro, arrebolamiento, sobresalto |
| Equilibrio | Lucidez | claridad, perceptividad, capacidad perceptiva |
|  | Estabilidad | saludable |
|  | Calma | apaciguamiento, bienestar, calma, levedad, pacificación, paz, quietud, reposo, serenidad, tranquilidad |
|  | Seguridad | seguridad, hogareño |
| Sentido del humor | Indulgencia | absurdo, clemencia, hilaridad, ingenio, ironía, ocurrencia |
| Enfado | Irritabilidad | adusto, gruñón, inquietud, malhumor, molesto, molestia |
|  | Exasperación | frustración |
|  | Rabia, ira, furia | amargura, animadversión, aversión, desprecio, enfado, ferocidad, furia, hostilidad, humillación, indignación, odio, rencor, resentimiento, venganza, violencia |
|  | Repulsión | asco, desprecio, repugnancia |
|  | Envidia | celos |
|  | Tormento | fastidio, molestia |
| Tristeza | Sufrimiento | agonía, angustia, culpa, dolor, herida, lástima |
|  | Aflicción | abatimiento, calamidad, congoja, depresión, desesperación, infelicidad, mal, melancolía, pena, pesadumbre, taciturno |
|  | Decepción, desilusión | arrepentimiento, consternación, desaliento, desolación, lamento, remordimiento |
|  | Abandono, negligencia, descuido | aislamiento, alienación, bochorno, derrotismo, humillación, injuria, inseguridad, insulto, ofensa, pesimismo, rechazo, repudio, retraimiento, soledad, vergüenza |
|  | Compasión | pena, piedad, conmiseración |
| Miedo | Horror | alarma, alteración, caos, conmoción, descontrol, espanto, flagelación, histeria, humillación, lástima, temor, mortificación, pánico, shock, susto, terror |
|  | Nerviosismo | ansiedad, aprehensión, disgusto, amedrentamiento, suspense, incertidumbre, desasosiego, preocupación |
| Vaciedad | Disminuido, empequeñecido | carente, carente de afecto, devaluado, incapaz, incompetente, incomprensión, insignificante, inútil/inutilidad, invisible/invisibilidad, poco importante, sin reconocimiento, sin valor, timidez |
|  | Insensible/insensibilidad | desconectado, disociado, escindido |
