# Supplementary material for: PROJECTA: An Art-Based Tool in Trauma Treatment
Source: Front Psychol. 2020 Dec 18;11:568948. doi: 10.3389/fpsyg.2020.568948 (PMC7775401; doi:10.3389/fpsyg.2020.568948)
Supplement: Supplementary file 3 [file Table_3.docx]

**Supplementary Material 3**

List of artistic works

Legend

* = artistic works that have been initially suggested by experts and have been chosen in the final list

In bold = works of art that belong to the final list but were not initially included by the experts, but in the second focus group

All other references = initial artwork suggested by experts

*Ader, B. J. (1971). *I'm too Sad to Tell You.* New York: MoMA*.*  <https://www.youtube.com/watch?v=KQ1U3XbEzR4>

AES+F (2007). *Tondo #24. From the Last Riot 2 series*.

*Agesandro, Atenodoro & Polidoro of Rhodes (27 d.C.). *Laocoonte and his sons.* Vatican City: Museo Pío-Clementino*.*  <https://commons.wikimedia.org/wiki/File:Laocoonte_and_their_sons.jpg>

Appel, K. (1954). *Caballos salvajes*. Madrid: Museo Nacional Thyssen-Bornemisza.

Armando (Herman Dirk van Dodeweerd) (1994). *Der Zaun*.

Australian Aboriginal Dot Paintings. *Art at Midway Middle School*. <http://2.bp.blogspot.com/-TB8boQcOyrs/Tk-47Y_wilI/AAAAAAAAAt0/cK-j6gC8QGk/s320/dot+painting.jpg>

Aziz + Cucher (1998). *Interior 1*, San Francisco: San Francisco Museum of Modern Art (SFMOMA)

Bacon, F. (1953). *Study after Velázquez's Portrait of Pope Innocent X.* Des Moines: Des Moines Art Center.

*Bacon, F. (1968) *Retrato de George Dyer en un espejo*. Madrid: Museo Thyssen-Bornemisza.<https://www.museothyssen.org/coleccion/artistas/bacon-francis/retrato-george-dyer-espejo>

Banksy (2002). *Girl with Balloon.* Shoreditch (Street Art)

*Berni, A. (1934). *Manifestación*. Buenos Aires: Museo de Arte Latinoamericano de Buenos Aires. <https://coleccion.malba.org.ar/manifestacion/>

Blanchard, M. (c. 1910-1915). *La española.* Paris: Musée d'Art Moderne de la Ville de Paris.

Blanchard, M. (c. 1912-1914). *Mujer con vestido rojo.* Santander: Museo de Arte Moderno y Contemporáneo de Santander y Cantabria (MAS)

*Borrell del Caso, P. (1874). *Huyendo de la crítica.* Madrid: Bank of Spain Headquarters*.* <https://commons.wikimedia.org/wiki/File:Escaping_criticism-by_pere_borrel_del_caso.png>

Botticelli, S. (c. 1477-1478). *The Spring/Allegoria della primavera.* Florence: Uffizi Gallery.

Bouguereau, W. A. (1895). *Le ravissement de Psyché.* Private collection.

*Bourgeois, L. (1986). *Articulated Lair*. New York: Museum of Modern Art (MoMA).<https://www.moma.org/collection/works/80872>

*Bourgeois, L. (1993). *Arch of Hysteria.* New York: Museum of Modern Art (MoMA).<https://historia-arte.com/obras/arco-de-histeria>

*Boyd, D. (2016). *Untitled (RW).*<http://www.idaia.com.au/en/events/guided-tours-indigenous-australian-art-mca-collection-and-biennale-of-sydney-2016/>

Brâncuși, C. (1910). *The kiss*. Philadelphia: Philadelphia Museum of Art.

**Cabellut, L. (1961). *Le Petit Prince*** [**https://orbecultural.wordpress.com/2017/01/20/lita-cabellut-los-conocimientos-nos-acercan-a-nuestras-pasiones-y-solamente-con-pasion-tenemos-una-oportunidad-de-ser-felices/**](https://orbecultural.wordpress.com/2017/01/20/lita-cabellut-los-conocimientos-nos-acercan-a-nuestras-pasiones-y-solamente-con-pasion-tenemos-una-oportunidad-de-ser-felices/)

**Cahn, M (1988). *Personen*, Nanjing: Sifang Art Museum.** [**http://www.artlinkart.com/en/artist/wrk_sr/793aAwlo**](http://www.artlinkart.com/en/artist/wrk_sr/793aAwlo)

Cao, M (n.s). *The red flower.*

*Caravaggio. (1597). *Medusa*. Florence: Uffizi Museum. <https://commons.wikimedia.org/wiki/File:Caravaggio_Medusa.JPG>

Caryatids from the south porch of the Erechtheion (c. 421-406 b. CE) Athens: Acropolis Museum

Cassatt, M. (1890). *Mujer con niño en brazos*. Bilbao: Museo de Bellas Artes de Bilbao.

*Chagall, M. (1915). *El cumpleaños.* New York: Museum of Modern Art (MoMA).<https://www.moma.org/collection/works/79360>

Chagall, M. (1917). *The Grey House.* Madrid: Museo Nacional Thyssen-Bornemisza

Coate, I. (no data). *Lorikeets in Love*. <https://iancoate.com/AF-Lori.jpg>

***Dalí, S. (1925). *Figura en una finestra*. Madrid: Museo Nacional Centro de Arte Reina Sofía.<https://www.museoreinasofia.es/coleccion/obra/figura-finestra-figura-ventana>

*Dalí, S. (1931). *The Persistence of Memory.* New York: Museum of Modern Art (MoMA).<https://www.moma.org/collection/works/79018>

Dalí, S. (1932). *Gradiva descubre las ruinas antropomorfas (Fantasía retrospectiva)*. Madrid: Museo Nacional Thyssen-Bornemisza.

*de Chirico, G. (1914). *The Melancholy and Mystery of a Street.* [Private Collection https://www.wikiart.org/en/giorgio-de-chirico/mystery-and-melancholy-of-a-street-1914](https://www.wikiart.org/en/giorgio-de-chirico/mystery-and-melancholy-of-a-street-1914)

de Goya, F. (C. 1797). *Mujer maltratada con un bastón*.

*de Goya, F. (c. 1797-1799). *El sueño de la razón produce monstruos.* Madrid: Museo Nacional del Prado*.*<https://artsandculture.google.com/asset/the-sleep-of-reason-produces-monsters-no-43-from-los-caprichos/FAF4YL0zP9cjHg?hl=es&ms=%7B%22x%22%3A0.5%2C%22y%22%3A0.5%2C%22B%22%3A8.017191811283963%2C%22z%22%3A8.017191811283963%2C%22size%22%3A%7B%22width%22%3A4.039349716446127%2C%22height%22%3A1.2375000000000007%7D%7D>

de Goya, F. (c. 1797-1799). *Sopla*. Madrid: Museo Nacional del Prado.

de Goya (1799). *Aquellos polvos*. Madrid: Museo Nacional del Prado.

de Goya, F. (1799). *Porque fue sensible.* Madrid: Museo Nacional del Prado.

de Goya, F. (1799). *Qué pico de oro.* Madrid: Museo Nacional del Prado.

*de Goya, F. (1814). *Los fusilamientos del 3 de mayo.* Madrid: Mueso del Prado.<https://www.museodelprado.es/coleccion/obra-de-arte/el-3-de-mayo-en-madrid-o-los-fusilamientos/5e177409-2993-4240-97fb-847a02c6496c?searchMeta=los%20fusilamientos>

*de Goya, F. (1820-1823). *Saturno devorando a su hijo*. Madrid: Museo del Padro.<https://www.museodelprado.es/coleccion/obra-de-arte/saturno/18110a75-b0e7-430c-bc73-2a4d55893bd6?searchMeta=saturno>

*de Goya, F. (1823). *Duelo a garrotazos.* Madrid: Museo Nacional del Prado.<https://www.museodelprado.es/coleccion/obra-de-arte/duelo-a-garrotazos/2f2f2e12-ed09-45dd-805d-f38162c5beaf?searchMeta=duelo%20a%20garro>

*de Goya’s disciple. *(1808-1812) El Coloso.* Madrid: Museo Nacional del Prado.<https://www.museodelprado.es/coleccion/obra-de-arte/el-coloso/2a678f69-fbdd-409c-8959-5c873f8feb82?searchMeta=el%20coloso%20goya>

de Lempicka, T. (1929)*. Self-portrait in a Green Bugatti.* Suiza: Private collection.

*de Lempicka, T. (1940). *La Fuite ou Quelque part en Europe*. Nantes: Musée des Beaux-Arts de Nantes.<https://www.pinterest.es/pin/530510031087357094/>

Degas, E. (1878)*. Dancers in green.* Private Collection.

Degas, E. (1878). *Two dancers in the studio (Dance School)*. Private Collection.

**Delacroix, E. (1830). *La Liberté guidant le peuple.* Paris: Musée du Louvre.** [**https://www.louvre.fr/en/oeuvre-notices/july-28-liberty-leading-people**](https://www.louvre.fr/en/oeuvre-notices/july-28-liberty-leading-people)

Delacroix, E. (1835). *Les natchez*. New York: The Metropolitan Museum of Art (MET).

*Delaunay, S (1915). *Market at Minho*. Private Collection.<https://arthive.com/soniadelaunay/works/379726~Mercado_de_minho>

*Denis, M. (1905-1906). *La corona de margaritas*. Madrid: Museo Nacional Thyssen-Bornemisza.<https://www.museothyssen.org/coleccion/artistas/denis-maurice/corona-margaritas>

di Bondone, G. (1302-1305). *The Massacre of the Innocents*. Padua: Scrovegni Chapel

*Donatello (c. 1453-1455). *María* *Magdalena.* Florence: Museo dell'Opera del Duomo.<https://commons.wikimedia.org/wiki/File:Maria_Magdalena,_Donatello,_Florencia,_Italia,_2019_01.jpg>

Ducreux, J. (1791)*. Portrait de l'artiste sous les traits d'un moqueur.* Paris: Louvre Museum.

Dumas, M. (1992). *The ritual (with doll)*. Tilburg: De Pont Museum of Contemporary Art.

Eisenman, N. (1992). *The minotaur hunt*. New York: Trial Balloon Gallery.

El Greco (c. 1576). *The Annunciation.* Madrid: Museo Nacional Thyssen-Bornemisza.

Erawan, N. (no data) *Unknown title.* <http://4.bp.blogspot.com/-Gsocr8UDJpI/UkA1P8pYVeI/AAAAAAAAAHM/nYR-El_WxD0/s1600/indonesian-artist-nyiman-erawan.jpg>

Ernst, M. (1926). *Young Virgin Spanking the Infant Jesus In Front of Three Witnesses.* Cologne: Museum Ludwig.

Fedorovna-Stepanova, V. (1920) *Billiard players*. Madrid: Museo Nacional Thyssen-Bornemisza.

Fini, L. (1949). *Angel of anatomy.*

Fini, L. (1964). *Heliodora*. Private Collection.

Flanagan, B. (1997)*. Hare (Thinker on a rock).* Washington D.C.: National Gallery of Art.

*Forner, R. (no data). *La ausencia*. Buenos Aires: Bonino Gallery

*Fragonard, J. H. (1767). *L'escarpolette*. Londres: Wallace Collection. <https://wallacelive.wallacecollection.org:443/eMP/eMuseumPlus?service=ExternalInterface&module=collection&objectId=65364&viewType=detailView>

Frederick, J. (1905). *Toms River*. Madrid: Museo Nacional Thyssen-Bornemisza.

Freud, L. (1976-1977). *Last Portrait.* Madrid: Museo Nacional Thyssen-Bornemisza.

*Friedrich, C. D. (1808-1810). *Monk by the Sea*. Berlin: Alte Nationalgalerie. . <https://commons.wikimedia.org/wiki/File:The_Monk_by_the_Sea_(Friedrich).jpg>

*Friedrich, C. D. (1818-1820). *A woman before the rising sun (a woman before the setting sun)*. Essen: Museum Folkwang.<https://commons.wikimedia.org/wiki/File:Caspar_David_Friedrich_-_Woman_before_the_Rising_Sun_(Woman_before_the_Setting_Sun)_-_WGA08253.jpg>

*Friedrich, C. D. (c. 1828-1830). *The Temple of Juno in Agrigento*. Dortmund: Museum Ostwall<https://commons.wikimedia.org/wiki/File:Agrigent_BW_2012-10-07_12-24-45.JPG>

*Gentileschi, A. (1610). *Susana y los viejos.* Bavaria: Schloss Weißenstein. <https://commons.wikimedia.org/wiki/Category:Susanna_and_the_Elders_by_Artemisia_Gentileschi,_Schloss_Wei%C3%9Fenstein#/media/File:Susanna_and_the_Elders_(1610),_Artemisia_Gentileschi.jpg>

*Gentileschi, A. (1612-1613). *Judith y Holofernes*. Napoli: Museo di Capodimonte.<https://commons.wikimedia.org/wiki/File:Judit_decapitando_a_Holofernes,_por_Artemisia_Gentileschi.jpg>

Gentileschi, A. (1620). *Jael y Sísara*. Budapest: Szépművészeti Múzeum.

*Géricault, T. (1819). *Le Radeau de La Méduse*. Paris: Louvre Museum.<https://commons.wikimedia.org/wiki/File:JEAN_LOUIS_TH%C3%89ODORE_G%C3%89RICAULT_-_La_Balsa_de_la_Medusa_(Museo_del_Louvre,_1818-19).jpg>

*Giacometti, A. (1961). *L’homme qui marche.* Pittsburgh: Carnegie Museum of Art.<https://historia-arte.com/obras/el-hombre-que-camina>

*Goldsworthy, A. (1982) *Balanced river stones Brough.*<https://www.goldsworthy.cc.gla.ac.uk/image/?tid=1982_136>

Hansen, J. (1952). [*Ali met groenteboer*](https://www.mutualart.com/Artwork/Ali-met-groenteboer/050443C1CEE4B2B4). New York: Christie's.

*Himid, L. (1985). *The carrot piece.* London: Tate Modern.<https://www.tate.org.uk/art/artworks/himid-the-carrot-piece-t14192>

Hokusai, K. (c. 1829-1833). *La gran ola de Kanagawa*. New York: Metropolitan Museum of Art (MET).

*Homer, W. (1872). *Retrato de Helena de Kay.* Madrid: Museo Nacional Thyssen-Bornemisza.<https://commons.wikimedia.org/wiki/File:Winslow_Homer_-_Portrait_of_Helena_de_Kay.jpg>

*Hopper, E. (1931). *Hotel Room.* Madrid: Museo Nacional Thyssen-Bornemisza.<https://www.museothyssen.org/coleccion/artistas/hopper-edward/habitacion-hotel>

*Hopper, E. (1942). *Nighthawks*. Chicago: Art Institute of Chicago.<https://www.artic.edu/artworks/111628/nighthawks>

*India (Punjab Hills, Kangra) (c.1800). *The Timid Bride.* New York: The Metropolitan Museum of Art (MET). <https://www.metmuseum.org/art/collection/search/37991>

*Izaguirre, G. (no data). *Circo 2*.

*Kahlo, F. (1944). *La columna rota*. Mexico D. F: Dolores Olmedo Museum.<https://artsandculture.google.com/asset/the-broken-column/EgGMbMFBQrAe3Q?hl=es&ms=%7B%22x%22%3A0.5%2C%22y%22%3A0.5%2C%22B%22%3A8.722202201418789%2C%22z%22%3A8.722202201418789%2C%22size%22%3A%7B%22width%22%3A3.47434493833709%2C%22height%22%3A1.2374999999999998%7D%7D>

*Katz, A. (2006). *Cow*. Boston: Museum of Fine Arts*.*<https://www.flickr.com/photos/ativandoneuronios/8739322611>

Kiefer, A. (2016). *Böse Blumen.*

*Kirchner, E. L. (1909). *Artistin (Marzella).* Berlin: Brücke-Museum. <https://es.wikipedia.org/wiki/Archivo:Ernst_Ludwig_Kirchner_-_Artistin_(Marzella).jpg>

Kirchner, E. L. (c. 1914). *Curving Bay.* Madrid: Museo Nacional Thyssen-Bornemisza.

***Klimt, G. (1907). *Danae*. Viena: Leopold Museum.<https://commons.wikimedia.org/wiki/File:Klimt_Danae.jpg>

*Klimt, G. (1910). *Death and Life*. Vienna: Leopold Museum.<https://commons.wikimedia.org/wiki/File:Gustav_Klimt_-_Death_and_Life_-_Google_Art_Project.jpg>

*Kollwitz, K. (1903). *Woman with her Dead Child/Frau mit totem Kind.* New York: MoMA*.*<https://es.wahooart.com/@@/8XYD5J-Kathe-Kollwitz-mujer-con-muerto-ni%C3%B1o>

Kollwitz, K. (1910). *Self-Portrait, Hand at the Forehead (Selbstbildnis mit der Hand an der Stirn).* New York: Museum of Modern Art (MoMA).

**Kollwitz, K. (1914). *Fear (Das Bangen) (in-text plate, p. 40) from the periodical Kriegszeit. Künstlerflugblätter, vol. 1, no. 10.* New York: Library of The Museum of Modern Art (MoMA)** [**https://www.moma.org/s/ge/collection_ge/objbyartist/objbyartist_artid-3201_tech-3_role-1_sov_page-3.html**](https://www.moma.org/s/ge/collection_ge/objbyartist/objbyartist_artid-3201_tech-3_role-1_sov_page-3.html)

**Kollwitz, K. (1920). *Woman Thinking.* Sydney: Art Gallery of New South Wales**[**https://www.artgallery.nsw.gov.au/collection/works/9351/**](https://www.artgallery.nsw.gov.au/collection/works/9351/)

*Kollwitz, K. (1931). *Madre e hijo/Mutter mit Jungen*. London: The British Museum<https://www.moma.org/collection/works/15910>

*Kollwitz, K. (1934). *Selbstbildnis (Self Portrait).* New York: MoMA*.*<https://www.flickr.com/photos/ativandoneuronios/8739322611>

Labille-Guiard, A. (1785). *Self-portrait with two pupils*. New York: The Metropolitan Museum of Art (MET).

Leonard, Z. (1995)*. Jennifer Miller does Marilyn Monroe.*

Leunig, M. (2018). *Ecosystem II.* Private Collection.

Liáng Kǎi (s. XIII). *Lǐ Bái (Li Po) reciting a poem*. Tokyo: National Museum of Tokyo.

Lion. Spanish mosaic (after 1200). New York: Metropolitan Museum of Art (MET)

*Ma Yuan (1160-1225). *Pesca apacible en un río durante el otoño.* Dinastía Song del Sur, China.<https://confuciomag.com/integracion-de-la-pintura-la-poesia-y-la-caligrafia-principios-esteticos-de-la-pintura-china-tradicional-vi>

*Magritte, R. (1928-1929). *La trahison des images* (*Ceci n’est pas une pipe).* Los Ángeles: Los Angeles County Museum of Art (LACMA).<https://collections.lacma.org/node/239578>

Mallett, K. (no data). *In mother hands*. <https://www.blackartdepot.com/products/in-mothers-hands-by-keith-mallett>

Mallett, K. (no data) *Sisters of the sun.* [*https://cdn.shopify.com/s/files/1/0217/9998/products/kema107.jpg?v=1492411710*](https://cdn.shopify.com/s/files/1/0217/9998/products/kema107.jpg?v=1492411710)

Mallo, M. (1939). *Canto de las espigas.* Madrid: Museo Nacional Centro de Arte Reina Sofía.

Mallo, M. (1941). *Cabeza de Mujer (frente).* Santa Fe: Museo Rosa Galisteo de Santa Fe.

Malyavin, F. A. (no data). *A laughing village baba.* Private collection.

*Mankes, J. (1913). *Sneeuwlandschap met sloot.* Netherlands: Museum Belvédere.<https://commons.wikimedia.org/wiki/File:Jan_Mankes,_Sneeuwlandschap_met_sloot,_Private_Collection_(Museum_Belv%C3%A9d%C3%A8re).jpg>

Master of the Saint Lucy Legend (c. 1475)*. Pietà Triptych.* Madrid: Museo Nacional Thyssen-Bornemisza.

*Matisse, H. (1909). *La Danse (I).* New York: MoMA.<https://www.moma.org/collection/works/79124>

Matisse, H. (1910). *La Danse* (II)*.* San Petersburgo: Hermitage.

Matisse, H. (c. 1910-1912)*. Les Capucines (Nasturtiums with The Dance II).* Moscow: Pushkin State Museum of Fine Arts.

*Messager, A. (1975). *La femme et…* Barcelona: Museu d’Art Contemporani de Barcelona (MACBA). <https://www.pinterest.es/pin/639089003341265310/>

Michelangelo (c. 1512). *The creation of Adam.* Vatican City: Sistine Chapel.

*Michelangelo (1547-1553). *The Deposition (The Florentine Pietà).* Floren ce: Duomo Opera Museum. <https://commons.wikimedia.org/wiki/File:Piedad_florentina.jpg>

*Michelangelo (1550). *The slave waking up.* Florence: The Accademia Gallery. <https://elpoderdelarte1.blogspot.com/2015/10/los-esclavos-de-michelangelo-buonarroti.html>

*Millais, J. E. (1851). *The Return of the Dove to the Ark.* Oxford: Ashmolean Museum.

*Millais, J. E. (1852). *Ofelia.* London: Tate Modern Museum.<https://commons.wikimedia.org/wiki/File:John_Everett_Millais_-_Ophelia_-_Google_Art_Project.jpg>

Millais, J. E. (1871). *The Martyr of the Solway.* London: Walker Art Gallery

Minjun, J. (no data). *Smiling face*. *Chinese LOL Selfies.*  <https://66.media.tumblr.com/00da5e45a4989777f514433e3ded08df/tumblr_nrgwgzfUsv1rv33k2o7_1280.jpg>

Miró, J. (1924). *Campesino catalán con guitarra.* Madrid: Museo Nacional Thyssen-Bornemisza.

Miró, J. (1925) *El Jardín.*

Miró, J. (1969). *La Harpie*.

Mithuna (s. VII). Detalle de escultura en arenisca del Templo de Lad Khan. Ahiole, India.

Modigliani, A. (c. 1917-1918). *Desnuda recostada.* Private collection.

Moholy-Nagy, L. (1921). *Circle Segments.* Madrid: Museo Nacional Thyssen-Bornemisza.

Mondrian, P. (1943). *Broadway boogie-woogie*. New York: Museum of Modern Art (MoMA).

***Monet, C. (1873). *Coquelicots.* París: Musée d’Orsay.<https://www.musee-orsay.fr/es/info/gdzoom.html?tx_damzoom_pi1%5BshowUid%5D=2278&tx_damzoom_pi1%5Bzoom%5D=1&tx_damzoom_pi1%5Bback%5D=%2F&cHash=be66a44ddf>

Monet, C. (1914-1917). *Water Lilies.* Canberra: National Gallery of Australia.

*Morandi, G. (1950). *Naturaleza Muerta.* Bologna: Istituzione Bologna Musei - Museo Morandi<https://arsmagazine.com/wp-content/uploads/2019/04/guggen-v.788.jpg>

*Moyse, E. (1872). *Inquisition.* New York: Jewish Museum. <https://thejewishmuseum.org/collection/1113-inquisition>

*Munch, E. (1893). *The Scream*. Norway: Munch Museum.<https://commons.wikimedia.org/wiki/File:Edvard_Munch_-_The_Scream_-_Google_Art_Project.jpg>

*Munch, E. (1896). *Separasjon*. Norway: Munch Museum.<https://commons.wikimedia.org/wiki/File:Edvard_Munch_-_Separation_(3).jpg>

Munch, E. (1900-1905). *Under the Stars*. Oslo: Munch Museum.

*Munch, E. (1906). *Self-Portrait with a Bottle of Wine*. Norway: Museo Munch.<https://es.m.wikipedia.org/wiki/Archivo:Edvard_Munch_-_Self-Portrait_with_a_Bottle_of_Wine_-_Google_Art_Project.jpg>

*Munch, E. (1907). *The Sick Child*. London: Tate Modern.<https://www.tate.org.uk/art/artists/edvard-munch-1678>

*Murakami, T. (2010). *Field of Smiling Flowers.*<https://paddle8.com/work/takashi-murakami/25923-Field-of-Smiling-Flowers/>

*Nieuwenhuys, C. (1950). *L'incendie.* Utrecht: Fondation Constant.<https://stichtingconstant.nl/work/lincendie>

*Nieuwenhuys, C. (1985). *L’insurrection.* Utrecht: Fondation Constant Nieuwenhuys. <https://stichtingconstant.nl/work/linsurrection-ii-0>

*Núñez, M. (1996). *Histérica roja*.<http://www.marinanunez.net/1996-galeria-8/>

*O’Keeffe, G. (1926). *Black Iris.* New York: MoMA<https://www.metmuseum.org/art/collection/search/489813>

Picasso, P. (1937). *Guernica*. Madrid: Museo Nacional Centro de Arte Reina Sofía.<https://www.museoreinasofia.es/coleccion/obra/guernica>

Picasso, P. (1937). *Mujer llorando.* Málaga: Museo Ruiz Picasso.

*Piccinini, P. (2010). *The comforter.* Sydney: Art Gallery of New South Wales<https://www.artgallery.nsw.gov.au/collection/works/309.2011/>

Pike, R. (no data). *Beana Lisa*.

*Quilty, B. (2006). *Joe*. <https://eyesing.typepad.com/eyesing/images/2007/11/27/7_2006_quilty_joe.jpg>

*Quilty, B. (2011). *Margaret Olley.* [Sydney: Art Gallery of New South Wales https://www.artgallery.nsw.gov.au/prizes/archibald/2011/28931/](https://www.artgallery.nsw.gov.au/prizes/archibald/2011/28931/)

Rai, R. (no data). *Untitled*. <https://www.tlcinteriors.com.au/wp-content/uploads/2015/10/Australian-Art-on-The-Life-Creative-Ruchi-Rai-Art.jpg.webp>

Rego, P. (no data). *Unknown Title.* Cascais: Casa das Historias. Paula Rego. (ENLAZAR IMG)

**Rego, P. (1998). *Untitled No.4. Abortion Series.* London: Tate Modern*.*** [**https://masdearte.com/lexico-familiar-paula-rego-la-virreina/**](https://masdearte.com/lexico-familiar-paula-rego-la-virreina/)

*Rembrandt (1630). *Self-portrait with wide open eyes and mouth shut.* Amsterdam: Rijksmuseum. .<https://www.rijksmuseum.nl/en/collection/RP-P-OB-697>

Renoir, P. A. (1876). *Bal au moulin de la Galette*. París: Musée d’Orsay.

Richardson, M. (no data) *Sumatran rhino*. <https://intlrhinofoundation.files.wordpress.com/2018/09/morgan-art-sumatran-rhino-updated.jpg?w=1472&h=1104>

*Rivera, D. (1941). *Flower Vendor (Girl with lilies).* California: Norton Simon Museum.<https://www.nortonsimon.org/art/detail/P.1980.2.3/>

***Rodin, A. (1882). *Le Baiser.* Londres: Tate Modern.<http://www.musee-rodin.fr/es/colecciones/esculturas/el-beso>

Rothko, M. (1960). *No. 14.*  San Francisco: San Francisco Museum of Modern Art (SFMOMA)

*Rothko, M. (1969). *Sin título.* Washington D.C.: National Gallery.<https://historia-arte.com/obras/negro-sobre-gris>

*Rousseau, H. (1910). *La Rêve.* New York: Museum of Modern Art (MoMA).<https://www.moma.org/collection/works/79277>

Sargent, J. S. (1904)*. Portrait of Millicent, Duchess of Sutherland.* Madrid: Museo Nacional Thyssen-Bornemisza.

Schippers, W. T. (1962). *Peanut-butter floor/Pindakaasvloer*. Amsterdam: Museum Fodor.

Serebriakova, Z. (1909). *At the Dressing-Table. Self-portrait.* Moscow: Tretyakov Gallery.

Sher-Gil, A. (1937). *Three Girls.* New Delhi: National Gallery of Modern Art.

*Sorolla, J. (1908). *Corriendo por la playa*. Oviedo: Museo de Bellas Artes de Asturias.<http://www.museobbaa.com/obra/corriendo-por-la-playa-valencia/>

Sorolla, J. (1909). *Chicos en la playa*. Oviedo: Museo del Prado. <https://www.museodelprado.es/coleccion/obra-de-arte/chicos-en-la-playa/edd7a202-c069-49f1-a3f4-eacf9b4022c2>

**Stern, G. (1949). *Los sueños de cansancio.*** [**https://www.pinterest.com.mx/pin/725079608731770722/**](https://www.pinterest.com.mx/pin/725079608731770722/)

**Stern, G. (1949). *Who Will She Be?*****https://www.newyorker.com/culture/photo-booth/grete-sterns-interpretation-of-dreams**

Stern, G. (1951). *Dream No. 28: Love Without Illusion*. Valencia: Institut Valencià d’Art Modern (IVAM).

Stom, M. (c. 1633 - 1639)*. The Supper at Emmaus.* Madrid: Museo Nacional Thyssen-Bornemisza.

**Sveinsson, A. (1944). *Helreiðin (stækkun).* Reykjavik: Ásmundarsafn.** [**http://www.fotosmundo.com/Europa/Islandia/Sudhurland/Reykjav%EDk/foto_32328.htm**](http://www.fotosmundo.com/Europa/Islandia/Sudhurland/Reykjav%EDk/foto_32328.htm)

**Tanning, D. (1943). *Eine Kleine Nachtmusik.* Tate Modern Museum, London.****https://www.tate.org.uk/art/artworks/tanning-eine-kleine-nachtmusik-t07346**

* Temple of Khajuraho, India (s. XI). *Mithuna*<https://commons.wikimedia.org/wiki/File:A_mithuna_scene_at_the_Le_Temple_Kandariya_Mahadeva_(Khajuraho).jpg>

*Unknown author. Detail of a figure of vihara I de Ajanta (s. V, India). Cuevas de Ajanta, India.<https://es.m.wikipedia.org/wiki/Archivo:Meister_des_Mah%C3%A2janaka_J%C3%A2taka_001.jpg>

Unknown author(s) (around 800). *Book of Kells/Leabhar Cheanannais.* Dublín: Trinity College Library.

Unkown author (Han dynasty. 206 a.C.-220 d.C. China). Figura de músico de un ajuar funerario. Chengdu: Xindu Museum.

Unknown author (I^st^ aC). *La poetisa de Pompeya.* Naples: National Archaeological Museum of Naples.

Unknown author (no data). *Erinyes.* <https://lapiedradesisifo.com/wp-content/uploads/2005/05/erinias.jpg>

Unknown author (no data). *Escaparate en Reikiavik* (expert's own file)

Unknown author (no data). *Niobe Myth*. <https://1.bp.blogspot.com/-vVrfecn8ios/VBSpfea4KDI/AAAAAAAAAVc/ToxYZOAYz-4/s1600/Niobe.jpg>

Unknown author (no data). Statue at the Georgia O'keeffe Museum (expert's own file). Santa Fe: Georgia O’keeffe Museum.

Unknown author (no data). *Vajrapani.* Tibet: White Temple of Tsapara.

Unknown author (no data). Title (monkey picking a fruit, indian ink). <https://www.google.com/search?sa=G&hl=es&tbs=simg:CAQSpgIJfJv1fxm29aoamgILELCMpwgaYgpgCAMSKLcSowbNArISqx22EswCsgedAvgSwSCHLpwu9C31LZsuiySJJOAswiAaMPfwLV7-aaOLDK77xyOMs3RI6JPrVPEC7S3tjmLZ77NlWzt4VMOlZtS6oeeHyeV1XCAEDAsQjq7-CBoKCggIARIEd6yy_1AwLEJ3twQkakgEKGQoGc2tldGNo2qWI9gMLCgkvbS8wN2dsenEKGwoJZ3JleWhvdW5k2qWI9gMKCggvbS8wM2QxMgojChFpdGFsaWFuIGdyZXlob3VuZNqliPYDCgoIL20vMDN5c3oKGQoHaGFycmllctqliPYDCgoIL20vMGZzZzgKGAoGY291Z2Fy2qWI9gMKCggvbS8wa2ZmMww&sxsrf=ALeKk01CDaRyugyhmiVA985mwchh6fKy1A:1588940672721&q=sketch&tbm=isch&ved=2ahUKEwiykPeioaTpAhXXiVwKHQRoCTkQwg4oAHoECA4QKA&biw=637&bih=623#imgrc=GEXIcs5KnnuBBM>

Unknown author (no data). Title. Exhibition of drawings (image from the expert's own file)

Vallotton, F. (1913). *The yellow sheet*. Private Collection

van Gogh, V. (1889). *The Starry Night.* New York: Museum of Modern Art (MoMA).

van Heemskerck, M. (c. 1531). *Portrait of a Lady spinning.* Madrid: Museo Nacional Thyssen-Bornemisza.

van Honthorst, G. (1624)*. The happy Violinist/Gelukkig violist.* Madrid: Museo Nacional Thyssen-Bornemisza.

*Van Wieck, N. (1990). *Q train.*<https://www.mundoflaneur.com/nigel-van-wieck-aires-de-hopper/>

Varo, R. (1955). *Simpatía.* Buenos Aires: Museo De Arte Latinoamericano De Buenos Aires (MALBA)*.*

***Varo, R. (1960). *Nacer de nuevo.* Ciudad de México: Museo de Arte Moderno. <https://remedios-varo.com/nacer-de-nuevo-1960/>

Varo, R. (1960). *Saliendo del psicoanalista.* México D. F.: *Museo de Arte Moderno.*

Vermeer, J. (1665). *The Girl With a Pearl Earring/Het meisje met de parel*. La Haya: Mauritshuis

Vordemberge-Gildewart, F. (1936). *Composition No. 104.* *White on White*. Madrid: Museo Nacional Thyssen-Bornemisza,

Waterhouse, J. W. (1903)*. Psique abriendo la caja de oro*. Private Collection.

Waterhouse, J. W. (1908)**.** *The soul of the rose.* Private collection.

Wesselmann, T. (1970). *Desnudo nº 1.* Madrid: National Museum Thyssen-Bornemisza.

Williams, S. (1991). *Try to be more accomodating*.

Wilson, M. (1974-2008). *I make up the image of my perfection/I make up the image of my deformity*.

Wouters, R. (1912). *Zote Geweld/Crazy Violence.* Amberes: Middelheim Museum

*Xiao, Lu (2003). *15 Shots: 1989-2003*. Virginia: The Fralin Museum of Art<http://www.howtotalkaboutarthistory.com/artist-feature/artist-feature-who-is-xiao-lu/>

Xul Solar, A. (1923). *Pareja.* Buenos Aires: Museo de Arte Latinoamericano de Buenos Aires (MALBA).

Yuon, K. (1921). *New Planet*. Moscow: Tretyakov Gallery.
